# Variations in vernacular naming of important species across three fishing villages of Chilika Lagoon, India

**DOI:** 10.1186/s13002-026-00848-x

**Published:** 2026-03-25

**Authors:** Natasha R. Serrao, Prateep Kumar Nayak

**Affiliations:** 1 School of Environment, Resources and Sustainability, University of Waterloo, 200 University Ave W, Waterloo, ON Canada; 2 School of Environment, Enterprise and Development, University of Waterloo, 200 University Ave W, Waterloo, ON Canada

**Keywords:** Ethnoichthyology, Chilika lagoon, Small-scale fisheries, Local ecological knowledge

## Abstract

**Background:**

Chilika Lagoon, India, is a biodiversity hotspot that is home to over 200 fish species and supports the livelihoods of over 400 000 fishers. A detailed record of fish names was previously undertaken; however, our initial field observations revealed differences in fish naming from those documented in the literature.

**Methods:**

This study examines local nomenclature of Chilika fishes in greater detail, with an emphasis on intra-cultural variation in fisher knowledge by taking an age-gender-village approach. Fieldwork was executed in three fishing villages across the lagoon by showing 56 colour photos of important fish to 108 local community members. Within each village, an equal number of respondents were selected across each of the three age groups and genders. The local name was documented for each fish, and the responses were analyzed according to age, gender, and village.

**Results:**

Across all photos, a total of 753 unique names were recorded, with many of these names attributed to phonetic differences. No notable age and gender differences in fish identification exist, except men were able to identify several fishes with higher success than women, and differences in fish naming exist across the three villages.

**Conclusions:**

This study revealed that the local names ascribed to Chilika fish were more extensive than documented in previous literature. This study emphasizes the importance of surveying basin-wide to capture the range of names associated with each fish species.

**Supplementary Information:**

The online version contains supplementary material available at 10.1186/s13002-026-00848-x.

## Introduction

Fishers possess substantial knowledge on fish ecology and behaviour, which is valuable for conservation, ecological restoration initiatives, and formulating fishery policies [1–3]. Adequately documenting linkages between vernacular and scientific naming of local fish is the first step in interpreting and applying fisher knowledge. The ability to link the local names to Linnean taxonomic nomenclature (scientific classification) allows us to accurately reference various plant and animal species despite the many names applied by different languages [4]. Additionally, the classification naming systems created and used by locals (folk taxonomy) indicate economic and social fish importance [5].

Jernudd & Thuan (1984) emphasize challenges that exist in our ability to use folk systems; these include inadequate knowledge about fish vernacular names, vernacular names are incorrectly recorded, and a lack of dissemination. Progress has been made over the last forty years to gain a better understanding of folk taxonomy within a local context; literature exists on vernacular fish naming by fishers via free-listing [5–7] and observations with fishers during boat surveys [8, 9]. However, these studies are not as numerous as their plant counterparts [10]; some studies that interview fishers even focus on fisher knowledge as it relates to plant species [11, 12].

Another consideration when conducting ethno-taxonomical research, is intracultural diversity due to age, gender, expertise, and social class [13–15]. Age-gender approaches have been widely applied within ethnobiology [14, 16, 17]; age and gender are both necessary to consider because they influence how people interact with biodiversity [14, 18, 19]. For example, while men are typically involved in the fishing activitiy itself, women play an invaluable role in the pre-and post-harvest activities, including sorting and fish trading [20]. Gender is reflected in the ability to free-list fish, and Renck et al. [15] found that men were able to free-list more fish than women; these findings are echoed in Gallois & Duda [21] and Aswani et al. [22]. With respect to age, ethnobiologists have observed that ecological knowledge increases with age as a result of increased opportunities and time to learn [14, 23], and in some cases, knowledge drops off after a certain age due to changes in responsibilities [24].

Previous studies have accounted for these social variables by showing the same fish photograph to different groups and recording their responses. These studies have spanned various countries including Argentina, Brazil, Malawai, Morocco, and Myanmar [25–29]. Across these studies, selections of photos were based on literature, photo availability, and fish that have been detected in a particular area [25, 28]. Fish species covered within these studies were as few as 24 [26] and as high as 218 [29]; in the latter case, two communities were surveyed with different sets of photos shown to each community. The number of these respondents ranged from 25 individuals [27] to 487 [25], and typically spanned all age groups; when surveying techniques were explicitly mentioned, fishers were selected via snowball, opportunistic, and based on 10 + years of activities [26–28]. Very few studies explicitly mentioned interviewing both men and women [28, 29]. Perspectives across all social and demographic variables are valuable because of the heterogeneity in knowledge that various fishers possess based on their identity [15, 30], and a systematic approach to documenting vernacular names could help achieve this.

Chilika Lagoon, India provides an opportunity to execute this type of research. The lagoon contains over 200 fish species, as well as 400 000 fishers that rely on the lagoon to support their livelihoods, and the gendered and class division (caste system) of labour. Based on this need, the primary aims of this paper are to examine: (i) the variation in vernacular naming of locally important fish in Chilika Lagoon, India, and (ii) any similarities and differences in local naming of various social groups (age, gender, village). Fishing occupation was customarily and historically pre-determined based on positioning within the Hindu Varna (caste) system [31]. Additionally, a comprehensive understanding of scientific to vernacular names already exist [32], and provides a starting point for this work. This study is the first in India to show photos to elicit vernacular names of locally important fish.

## Methods

### Study location

Chilika Lagoon is situated on the Eastern coast of Bay of Bengal, and is located between the longitude 85°05’ to 85°38’E and latitude of 19°28’ to 19°54’N [33]. Chilika Lagoon spans three administrative districts in Odisha state including Puri, Ganjam, and Khurda [34]. There are 52 tributaries that drain into Chilika Lagoon, with the water depth of the lagoon ranging from 0.38 to 4.9 m based on season, while the area ranges from 704 km^2^ to a maximum of 1020 km^2^ [33, 34]. It is home to 225 fish, 35 crab, and 28 shrimp/prawn known species, many of which are endemic, and contains a combination of marine, freshwater and brackish water species [33].

### Study sites

**Fig. 1:**
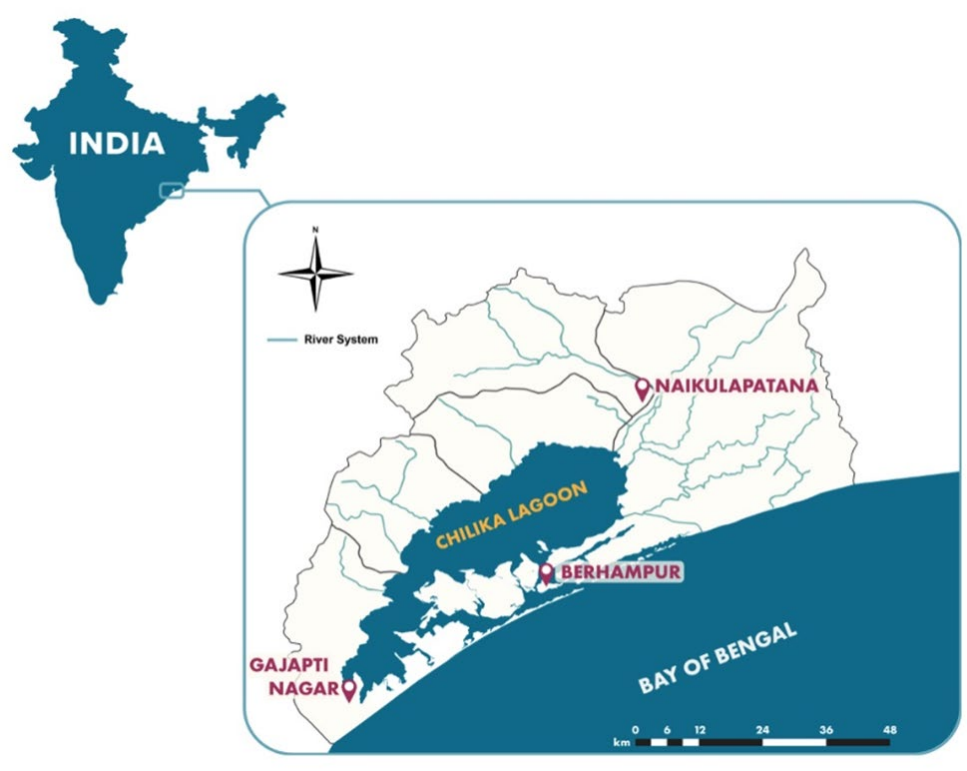
Map of Chilika Lagoon, showing the three fishing villages selected for fish photo identification in June 2023

We conducted a fish photo identification exercise in three fishing villages within Chilika Lagoon during June 2023. Villages chosen for this study included Berhampur, Naikulapatana, and Gajapati Nagar (Fig. 1). See Table 1 for site characteristics. The criteria applied for village selection included: i) comprised primarily of fishers, (ii) a different caste category is prominent in each selected village, (iii) geographically dispersed, and (iv) proximal to a major water feature (sea, river, canal).

### Fish photo identification survey interviews

We developed an in-person interview protocol using the following steps (we refer to this as an “interview survey” [25]). First, we compiled a list fish of species via household surveys across all three villages (*n* = 54) based on vernacular names, with 30 households selected through random sampling in each village. This list was based on overall importance, as well as for eating, selling, festivals, marriages, community gatherings and caste. Each fish was cross-referenced to obtain Latin and English names using species identifications in Suresh et al. [32]. The photo associated with each fish was extracted from Suresh et al. [32], and a PowerPoint presentation was generated. Each slide consisted of a coloured photo of one fish and its associated numeric code; the presentation was viewed by the fishers on a laptop computer.

In twelve separate cases, multiple species were given the same vernacular name (see Supplementary Material 1). To select a representative fish for each of these twelve cases, we showed all photos of all possible species within a group to the fishers. They were asked to (i) provide a vernacular name for each fish and (ii) identify the most common fish within that group. We then cross-referenced the fish in Suresh et al. [32] to confirm the most common species so that it had the highest chance of being recognized across all villages. Part (i) was carried out across all three villages, while part (ii) was carried out in just Berhampur; we could not visit all three villages before selecting a representative fish. For example, the name ‘khuranter’ (note; all vernacular names in Odiya language will be accompanied by single quotation marks) contains multiple species including ‘dhala khuranti’ (*Rhabdosargus sarba)*, ‘kala khuranti’ (*Acanthopagrus longispinnis)*, ‘kala khuranti’ (*Acanthopagrus berda)*, and ‘haribolia khuranta’ (*Crenidens crenidens).* Within Berhampur, all four fish were identified as some variation of ‘khuranter’, with *Rhabdosargus sarba* being selected as the most common among Berhampur fishers, as well as found abundantly throughout the lagoon [32]. As such, this fish was selected as a representative species to be used in the 56 slides.

The 56 photos representing 54 species were shown to Chilika fishers in three sampled villages. Two photos each of species 'rohi' (*Labeo rohita*) and 'khainga' (*Mugil cephalus*) were shown to villagers. The species covered a total of 15 orders including Anguilliformes (2), Beloniformes (3), Carangiformes (1}, Characiformes (1), Clupeiformes (4), Cypriniformes (8), Elopiformes (1), Gonorynchiformes (1), Mugiliformes (8), Myliobatiformes (1), Osteoglossiformes (1), Perciformes (17), Pleuronectiformes (1), Siluriformes (6), and Tetraodontiformes (1).

The photo identification interview survey was applied across the three villages using a random stratified sampling approach. Fishers were stratified based on age groups (25–48, 49–72, 73–96) and gender (male or female) in each village, with six individuals selected per stratum. Convenience sampling was undertaken for Naikulapatana for males across all age groups and female seniors due to limited availability of contributors because of migration of young and middle-aged men, and a small sample size for seniors. A total of *n* = 108 interview surveys were completed across all three villages (*n* = 36 interview surveys per village). See Table 1 for demographic profiles of the respondents. All interview surveys were completed jointly with a community researcher from the Chilika (local) fisher community, which was critical to navigate the cultural and language barriers.

**Table 1:** Sociodemographic characteristics of interviewed fishers in Chilika lagoon

	Berhampur	Naikulaptana	Gajapati Nagar
Ethnicity	Indian	Indian	Indian
Language	Odiya	Odiya	Odiya
Religion	Hindu	Hindu	Hindu
Predominant fisher caste	Kaibartya and Khatia	Kaibartya	Kandara
District location	Puri	Puri	Khorda
Channel location	Outer	Northern	Southern
Number of inhabitants on voter list	1549	936	2000
Interviewees’ age *equal men and women interviewed in each age group			
25–48	12	12	12
49–72	12	12	12
73–96	12	12	12
Ecological significance	Proximity to Bay of Bengal sea mouth (Island)	Upstream of Daya river mouth	Proximity to lagoon and Palur canal
Water type	Brackish to marine	Freshwater	Brackish to freshwater
Gear type	Fixed gear (screen barriers (predominantly) and drag nets (less frequent))	Mobile (gill and trammel nets) and fixed gear (screen barriers)	Fixed gear (screen barriers)
Fishing rights	Lottery system close to island	Fish in free areas within lagoon	Fish in areas subleased by village
Role of men in fishing	Men return home daily and fish for few hours at a time	Typical to leave days to weeks at a time	Returns home daily and fish for a few hours at a time
Role of women in fishing	Do not typically enter lagoon. Separate fish catch, clean, cook fish. Can also include selling fish to trader and dry fish processing	Do not typically enter lagoon. Clean and cook fish	Women can enter the lagoon to support their husband with fishing activities when needed. Undertake additional labour roles to supplement household income

Interview surveys were conducted with contributors at their homes to minimize outside interference. Once verbal consent was obtained, we showed the contributor a photo and asked them to name the fish species. This was repeated 55 times per interview (once for each of the 56 important fish species) using the Powerpoint Presentation, for the compiled list of important fish species. In addition to the audio recording, responses were written on the data sheet (see Supplementary Material 2), and age, gender, and caste information for each contributor was also acquired. Questions were primarily limited to the fish identification; there were very few cases of follow-up questions being asked. A response of “pass” indicated that the contributor could not recognize the fish, or that they recognized the fish but could not assign a name. This was then classified under “not recognized”. When multiple people were in the room for the interview survey, we requested that only the contributor participate in the interview survey. Although minimal, it was at times challenging to mitigate bias because of outside influences when providing vernacular fish names; in these cases, the final response of the contributor was recorded.

For our research in Chilika Lagoon, ethics clearance for research with human contributors was obtained from the University of Waterloo (#44495) and followed when completing surveys; this includes informed consent, confidentiality, permission to record audio, and respect.

### Fish photo identification data analysis

Fish vernacular names were transcribed in English by an Odiya speaking co-author (PKN) whose mother tongue is also Odiya, so that variations in fish names could be captured. Partial audio files were missing for one interview survey, and as such, the responses written by the English-speaking co-author were applied. Each contributor’s responses across the 56 photos were imported into Excel to create a master sheet of answers for analysis in R-studio. The *openxlsx*, *tidyverse* and *dbplyr* packages in R-studio were used to group local names for each photo according to strata (village, gender, age grouping). Direct quotes were taken from audio recordings to support the study findings.

As a result of the large variation in local names assigned to each photo, we created a validation step to classify each name as “accepted”, “uncertain”, or “not recognized”. A local name was listed as “accepted” if it was either an exact match or minor variation to the name listed in Suresh et al. [32] and was verified by researchers. A local name was listed as “uncertain” if the name was not listed in Suresh et al. [32] *or* there were three or more variations of a given name and/or were five or more guesses for a particular name variant. A local name was listed as “not recognized” if the family classification did not match with the family classification of the given name *or* there were less than three variants of a given name *and/or* less than five guesses for a particular name variant. (see example below; Table 2). To help group these names, we used the following levels as a protocol: (i) family and genus taxonomic classifications, (ii) textbook and document check, (iii) consultation with resource people in Chilika.

**Table 2:** Example of validation step for ‘dhala khuranti’ (goldlined seabream, *Rhabdosargus sarba*)

Accepted Name	Not recognized	Uncertain
Khurandi (1)	Kanti (1)	Chandi (12)
Khuranta (40)	Pass (Madhura) (1)	Changuna/Chandi (1)
Khuranti (25)	Pass (8)	Desi Chandi (1)
		Dhala Jagili (1)
		Jagala (7)
		Jagali (2)
		Jageli (1)
		Jagili (7)

Responses under each column represent the vernacular name assigned to each category. Numbers in brackets correspond to number of local community responses for the vernacular name

Pearson’s Chi-Square tests (α = 0.05) were run in R to compare observed frequencies to those expected to determine if differences in naming across village, gender, and age are the result of a true relationship or chance. Six photos were removed from analysis for the village test, while five were removed from gender and age because the expected values were less than 5. Vernacular names listed as “accepted” were assigned “1”, while those listed as “uncertain” or “not recognized” were assigned “0”.

## Results

### Variation in vernacular naming

**Fig. 2:**
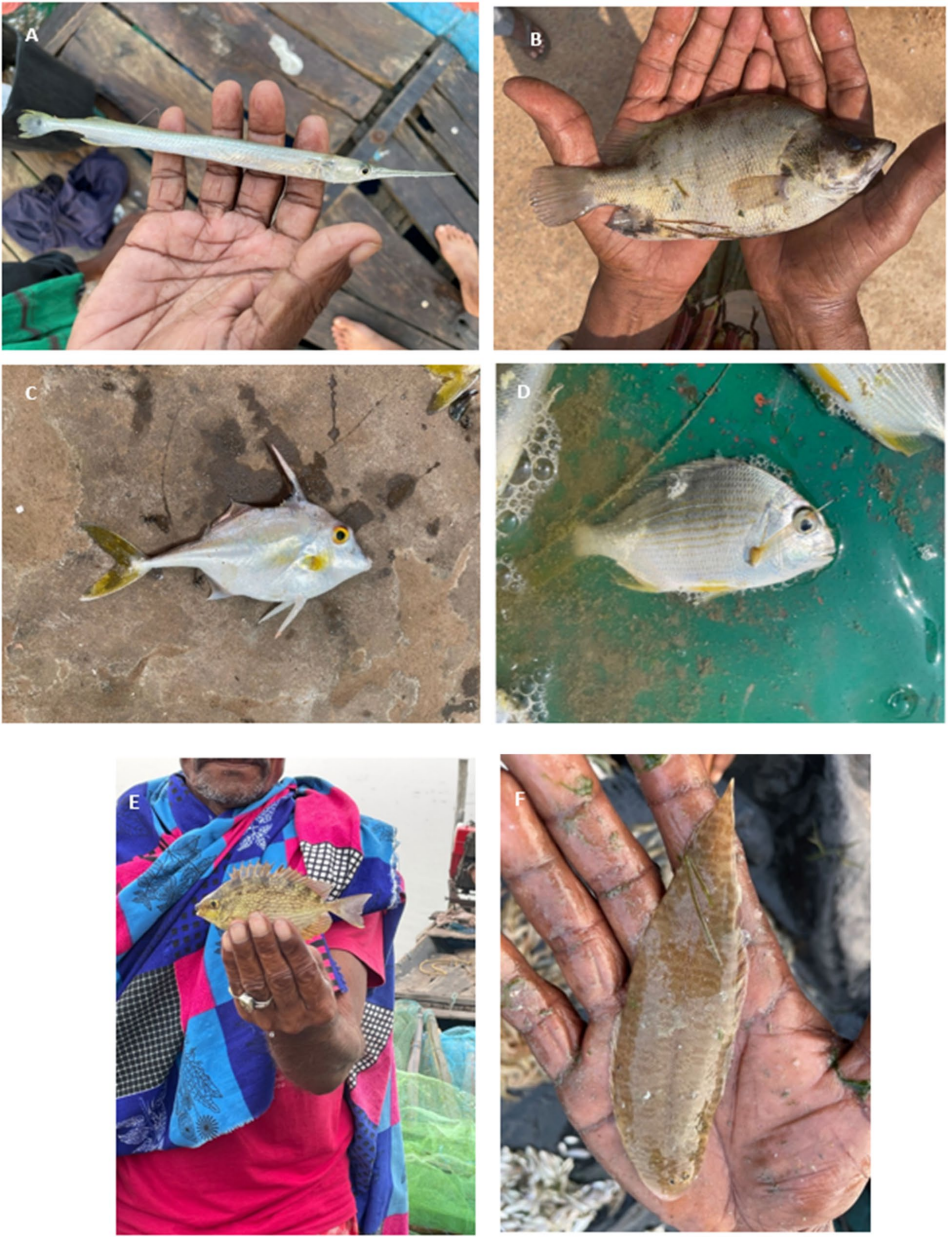
Photos of local Chilika fish: **A**) **‘**gania’ (spottail needlefish, *Strongylura strongylur)*, **B**) ‘verenda/udari’ (silvertiger perch, *Datnioides polota*), **C**) ‘sukura’ (short-nosed tripodfish, *Tricanthus biaculeatus*), **C**) ‘dhala khuranti’ (goldlined seabream, *Rhabdosargus sarba*), **E**) ‘samadho’ (streaked spinefoot, *Siganus javus*), **F**) ‘aswa’ (speckled tongue sole, *Cynoglossus puncticeps*)

These 54 species elicited a total of 753 local names, with the most common vernacular name being “sea fish”. For clarity, we listed species by common names, with scientific names written in brackets. There was an average of 25 names per photo and a median of 26 names per photo. Results from this study revealed a high diversity of names assigned to many species. Across the 56 photos, the number of local names per photo ranged from 6 to 44, with ‘gania’ (needlefish, *Strongylura strongylura*; Fig. 2a) having the lowest number of names and ‘verenda/udari’ (silvertiger perch, *Datnioides polota*; Fig. 2b) having the highest number of names (see Supplementary Materials 3 and 4 for a breakdown of responses based on local name and villages).

A significant portion of the diversity observed is attributable to minor pronunciation differences in vernacular names. For example, across all villages, *Tricanthus biaculeatus* (short-nosed tripodfish; see Fig. 2c) is given the names ‘sakura’, ‘sukuda’, ‘sukura’, and ‘sukuta’, while *Rhabdosargus sarba* (goldlined seabream; see 2d) is given the names ‘khurandi’, ‘khuranta’, ‘khuranti’. Even within a village, variations in vernacular naming can exist. *Eleutheronema tetradactylum* (fourfinger threadfin) is given the names ‘baisiali’, “baiya (Sahali)”, ‘baya’, ‘baya pilla’, ‘sahala’, ‘sahali”, “sahalia”, “shahali” within Berhampur. In some instances, fishers assigned different names to the fish based on perceived size differences. For example, the same photo of *Gibelion catla* (Indian major carp) elicited a wide range of responses including “bhakura”, “bhakura chhua”, “bhakura pilla”, wherein chhua and pilla refer to smaller sizes. The same is true of *Labeo rohita* (Indian major carp), which had responses including “rohi”, “rohi chhua”, “rohi pilla”, “rohia”.

For each photo, at least one or more individuals across the 108 contributors had a “pass” response. The four fish with the lowest number of passes were ‘Chilika khainga’ (flathead mullet, *Mugil cephalus*) and ‘menji’ (Otomebora mullet, *Planiliza melinopterus*) with one pass, and ‘singada’ (threadfin sea catfish, *Arius arius*) and ‘parsi soradi’ (goldspot mullet, *Chelon parsia*) with two passes. The four fish with the highest number of passes were ‘nadiakhai kokoli’ (slender rainbow sardine, *Dussumieria elopsoides*) and ‘roopchandi’ (pirapitinga, *Piaructus brachypomus*) with 41 passes, ‘chalanta/phula kerandi’ (peninsular Osteobrama, *Osteobrama peninsularis*) with 45 passes, and ‘khursia’ (kuria labeo, *Labeo gonius*) with 51 passes. The most populous local name assigned across all photos included ‘chandi’ (order: Perciformes) (17 photos), ‘khuranti’ (family: Sparidae) (16 photos), and ‘kabala’ (flathead mullet, *Mugil cephalus*) (15 photos). The photo of fish *Cynoglossus puncticeps*, identified as ‘aswa’ (speckled tongue sole) by the textbook, did not match the identification provided by the villagers; this was the only instance where there was no congruency in village and textbook identification. Instead, variations of the name ‘patmacha’ were popular among Naikulapatana fishers (25/36), while ‘patua’ was popular among Berhampur (16/36) and Gajapati Nagar fishers (21/36).

### Age, gender and geography differences in fish identification

**Fig. 3:**
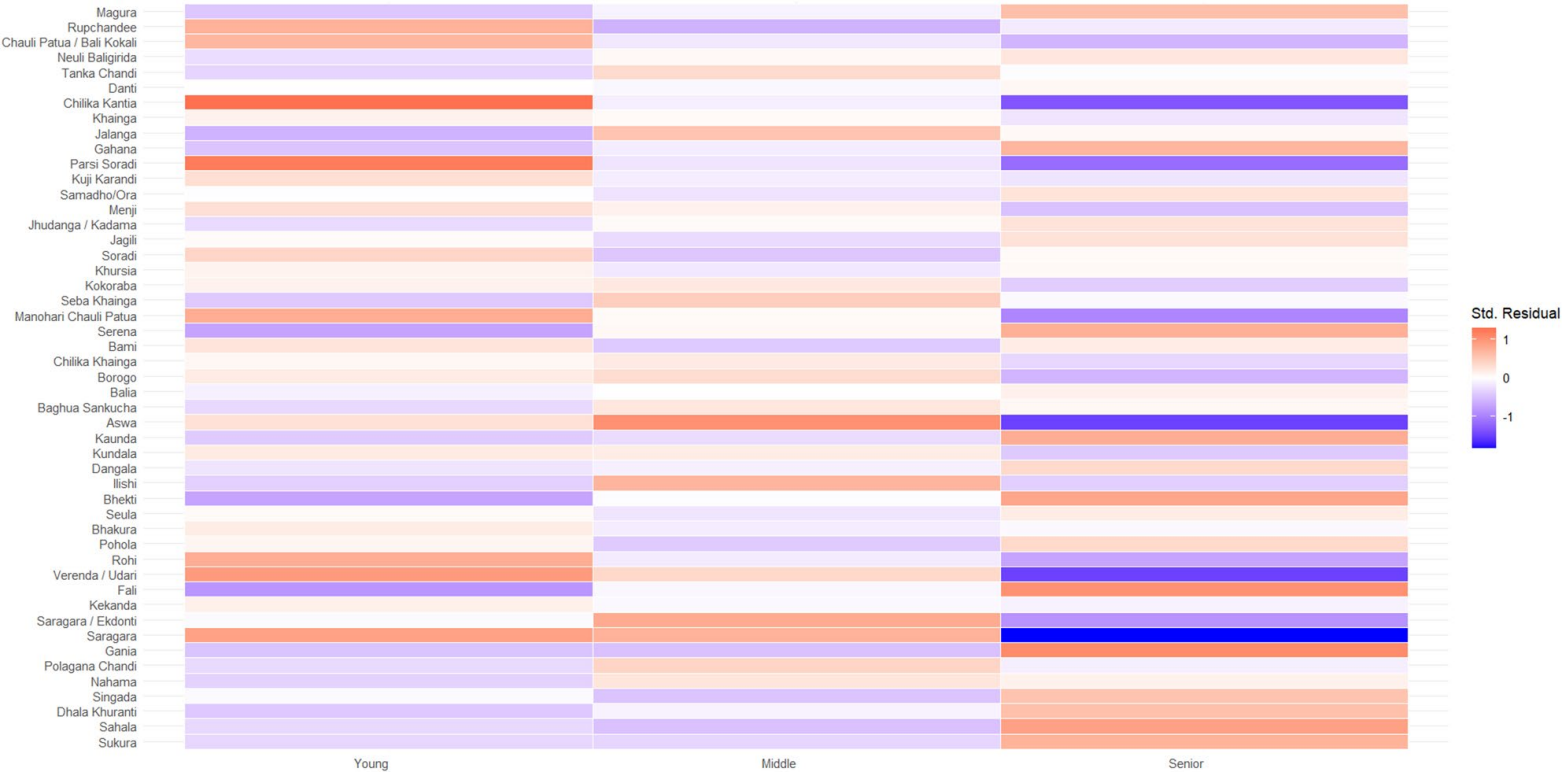
Residuals from Chi-Square test with x-axis representing age (young, middle, senior) and y-axis representing photo of fish shown to fisher. Darker red represents over-representation, in that observed values are greater than expected values, while darker blue represents under-representation, in that expected values are greater than observed values

**Fig. 4:**
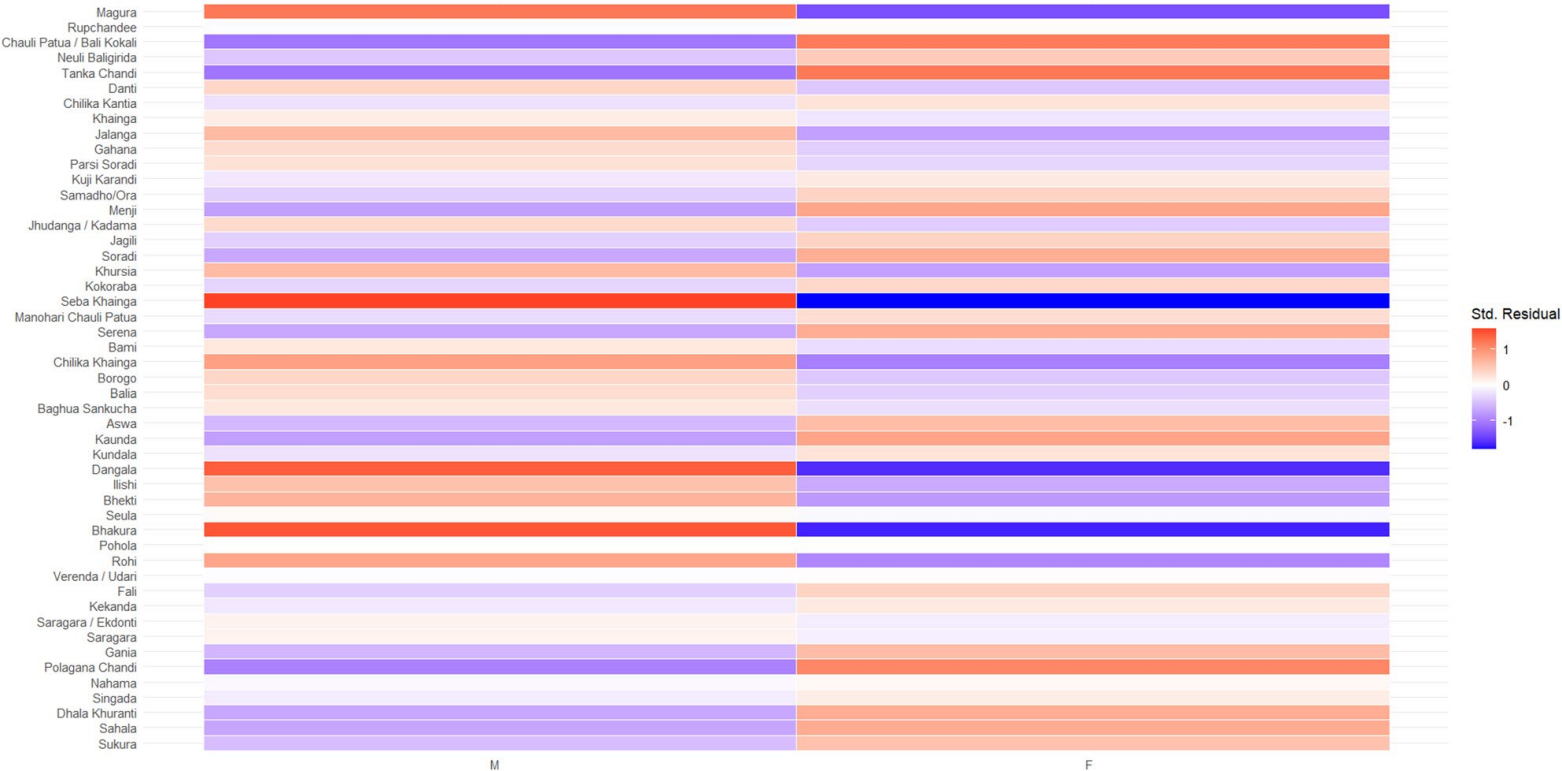
Residuals from Chi-Square test with x-axis representing gender (male (m) and female (f)) and y-axis representing photo of fish shown to fisher. Darker red represents over-representation, in that observed values are greater than expected values, while darker blue represents under-representation, in that expected values are greater than observed values

There were no significant associations between fish naming and age (χ^2^ = 41.094, df = 100, *p* = 1) and gender (χ^2^ = 46.048, df = 50, *p* = 0.633) across all photos; see Figs. 3, and 4 for residuals of chi-square test. Here, we define success as congruence between the vernacular names assigned by Suresh et al. [32] and that of the fishers. Except for ‘bhekti’ (Asian seabass, *Lates calcariferi*) and ‘borogo’ (Bengal corvine, *Daysciaena albida*), there were no notable gender differences in how fishers identified the species. However, across most age groups and villages, males were able to identify ‘bhekti’ and ‘borogo’ with higher success than females. An observation from audio recordings was that females sounded more confident than males in their responses. The small sample sizes coupled with the large variation observed within each stratum and ambiguities in vernacular names hindered the ability to draw meaningful conclusions.

Villagers demonstrated some difficulties recognizing and identifying fish beyond their fishing range. Berhampur and Gajapati Nagar fishers were able to identify ‘kekenda’ (corsula mullet, *Rhinomugil corsula)*, ‘fali’ (bronze featherback, *Notopterus notopterus)*, and ‘udari/verenda’ (silver tiger perch, *Datnioides polota)* with limited success, while Naikulapatana fishers were able to identify the same fish with high success. For example, ‘kekenda’ was identified correctly by 81% (29/36) of contributors in Naikulapatana, 8% (3/36) in Berhampur, and 14% (5/36) in Gajapati Nagar. Here, correctly means in agreement with Suresh et al. [32]. In another example, ‘fali’ was identified correctly by 100% (36/36) of contributors in Naikulapatana, and only 11% in Berhampur (4/36), and 47% (17/36) in Gajapati Nagar. Similarly, ‘udari/verenda’ was identified correctly by 61% (22/36) or (79% (20/24) with senior fishers excluded) of contributors in Naikulapatana, zero contributors (0/36) in Berhampur and 8% (3/36) of contributors in Gajapati Nagar. Note, Naikulapatana fishers only used variations of ‘udari’, while Gajapati Nagar fishers only used variations of ‘verenda’. Similarly, Naikulapatana fishers were the only ones able to name ‘pohala’ (reba carp, *Cirrhinus reba)* (83% or 30/36), with no one in Berhampur and Gajapati Nagar being able to identify this fish. Lastly, in the example of *Siganus javus*, identified as ‘samadho’ by Suresh et al. [32], most fishers in Berhampur (33/36) identified this fish as such, whereas ‘ghira’ was the prominent name in Gajapati Nagar (25/36). This fish was identified as some variation of ‘potala’ (11/36) in Naikulapatana.

**Fig. 5:**
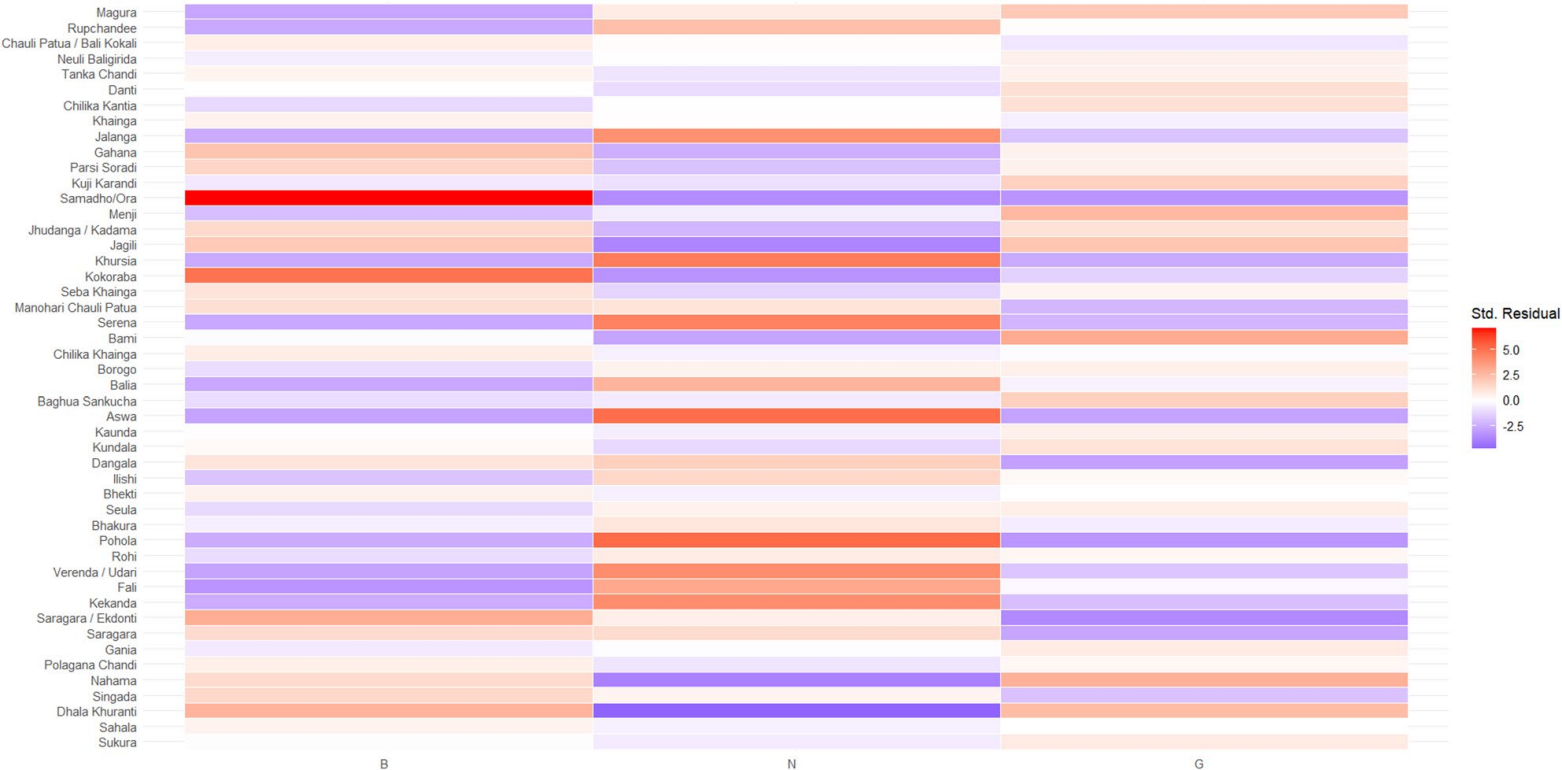
Residuals from Chi-Square test with x-axis representing village (Berhampur (B), Naikulapatana and Gajapati Nagar (G)) and y-axis representing photo of fish shown to fisher. Darker red represents over-representation, in that observed values are greater than expected values, while darker blue represents under-representation, in that expected values are greater than observed values

While different communities often attributed the same names to fish (see Supplementary Material 3), large variations in vernacular naming also exist between communities. There was a significant association between fish naming and village (χ^2^ = 673.62, df = 98, p = < 0.05; see Fig. 5). For example, over 88% of Berhampur (33/36) and Gajapati Nagar (32/36) fishers identified ‘khuranta’ (goldlined seabream, *Rhabdosargus sarba*) as some variation of ‘khuranta’, while over 88% of fishers in Naikulapatana named this fish as a variation of either ‘jagili’ or ‘chandi’. Jagili (*Gerres filamentosus*) was identified as such by over 70% of fishers in Berhampur (26/36) and Gajapati Nagar (27/36), while only 11% (4/36) of Naikulapatana fishers identified it as some variation of ‘jagili’, and 61% (22/36) of fishers identified it as some variation of ‘chandi’. ‘Tanka chandi’ (*Leiognathus equulus*) was identified as ‘chandi’ by over 75% of fishers across all villages. Similarly, a photo of *Siganus javus* (see Fig. 2e) was shown to Chilika fishers with vernacular name assigned as ‘samadho/ora’ by Suresh et al. [32]. Within Berhampur, 91% (33/36) of fishers identified this fish as a variation of ‘samadho/ora’, while no fishers in Naikulapatana or Gajapati Nagar identified it as such. Within Gajapati Nagar, 70% (25/36) of fishers named this fish as some variation of ‘ghira’, while 31% of fishers named this as some variation of ‘potala’.

## Discussion

This study represents the first of its kind in Chilika Lagoon, India to understand patterns in vernacular naming conventions for locally important fish species, with the contributions from 108 fishers across three villages. This study builds on the work by Suresh et al. [32] by factoring in basin-wide perspectives in cataloguing local names of harvested species; here we account for sensitivity to ecological and geographic differences within the basin. Results from this study revealed a high diversity of vernacular names assigned to each species, with many of these names being attributed to small phonetic differences within and among communities. In cases where species did not occur across all villages, accurate fish identification was higher in villages where fish are caught, re-affirming the need to undertake basin-wide perspectives.

### Vernacular variation within and between communities

A major finding from this study is the high diversity of names assigned to each species. These findings parallel those from other studies; Ambali et al. [25] found an average of 10.2 local names assigned to each fish photo in Lake Malawi, while Masski & Hammou [27] found a median number of nine vernacular names for each species. The seminal work undertaken by Suresh et al. [32] generally assigned one Odiya name to each fish species, and therefore, did not capture the diversity of vernacular names. Reporting of some local names might be accompanied by a slight inflation, as the name could have different adjectives with similar meanings; for example, ‘bhakura chhua’ and ‘bhakura pilla’ both refer to small sizes of ‘bhakura’ (Indian major carp, *Gibelion catla*). The examples of ‘dhala khuranta’ (*Rhabdosargus sarba*) and ‘sakura’ (*Tricanthus biaculeatus*) illustrate the range of names assigned to a singular species, with a major factor being attributable to pronunciation variances, similar to the small phonetic differences observed in the vernacular naming of Moroccan and Northeast Brazilian fish [15, 27]. This variation is also prevalent within Chilika-specific published literature, where *Notopterus notopterus* is named ‘fali’ [32] and ‘flai’ [35], *Wallago attu* is named ‘balia’ [32] and ‘balhia’ [35], and *Cirrhinus reba* is named ‘pohola’ [32] and ‘pohala’. The vernacular variations (both crude and subtle), highlight the need to engage local researchers in data interpretation and analysis; without this, slight phonetic variations might not be accurately captured, and there exists a possibility of incorrectly interpreting data gathered [36]. This diversity is also evident within the FishBase database, wherein many vernacular names exist globally for each species of fish (see Supplementary Material 5).

This study sought to uncover variations in vernacular naming of fish in Chilika in disparate communities and found differences in fish naming across villages. Chilika fish ‘kekenda’, ‘fali’, ‘udari’ and ‘pohala’ were identified with high success by Naikulapatana fishers and limited success by Berhampur and Gajapati Nagar fishers. These fish are primarily freshwater in nature and/or reside within the Northern Region [32], and therefore posed challenges in identification for Berhampur and Gajapati Nagar fishers. These nuances in fish identification across communities, potentially related to fishers not actively catching target species, are critical to ensure that information is disseminated accurately to fishers and management authorities within these communities [37]. Other reasons could include ecological and habitat type, proximity to sea or river mouth, influence of market and the value of the fish, local preferences associated with the fish in terms of cultural significance, taste, and more. If these differences are not captured, they could lead to fish misidentification or ambiguity [5, 38].

From previous studies, it has been observed that each fishing community creates their own unique ethnotaxonomic system, and while names can be consistent across communities, variations can also exist [5]. Across all three villages, ‘aswa’ was not assigned to *Cynoglossus puncticeps* by any of the locals (see Fig. 2f). Instead, the vernacular name ‘patamacha’ ascribed by Baliarsingh et al. [35] was prominent with Naikulapatana fishers, and a variation of ‘patua’ was prominent with Berhampur and Gajapati Nagar fishers. ‘Dhala khuranti’ was identified as ‘jagili’ by Naikulapatana fishers, while all other variants of ‘khuranti/khuranta’ were identified as ‘khuranta’ during the group interviews (the purpose of which was to select a representative fish when multiple species exist for a vernacular name; see Supplementary Material 1). In the example of *Siganus javus*, identified as ‘samadho’ by Suresh et al. [32], most fishers in Berhampur identified this fish as such, whereas ‘ghira’ was the prominent name in Gajapati Nagar and ‘potala’ was the name given in Naikulapatana, albeit less frequently. These responses were also reflected in the initial group interviews (see Supplementary Material 1). Similarly, *Gerres filamentosus*, identified as ‘jagili’ by Suresh et al. [32], was identified as such by most fishers in Berhampur and Gajapati Nagar , whereas ‘chandi’ was the prominent name in Naikulapatana. It was later inferred that Naikulapatana fishers classify ‘jagili’ as a type of chandi; when asked about the availability of ‘chandi’, the following conversation between a fisher and the research assistant ensued:

*Research assistant: in what months is Chandi available?*

*Fisher: What kind of Chandi? There are four types of Chandi.*

*Research assistant: Chilika Chandi.*

*Fisher: One is Tanki Chandi*,* Ghee Chandi*,* Jagili.*

*Research assistant: No, Jagala is Jagala*

*Fisher: Chandi*

Additionally, when the group interviews were undertaken, the majority of Naikulapatana fishers identified all variants of ‘jagili’ as ‘chandi’, while few put forward the name ‘jagili’.

Cases exist of different vernacular names being assigned to perceived size differences in a fish species. For example, Jones & Sujansingani [39] identify ‘sahal’ (large), ‘sahalia’ (medium), and ‘baisali’ (small) for species *Eleutheronema tetradactylum*; all three names were captured across the villages within my study, in addition to other vernacular names including ‘sahala’, ‘sahali’, ‘sahalia’. This parallels research findings from Pinto et al. [5], who reported that ‘pema’ was the vernacular name given to the fingerlings of *Megalops atlanticus*, while ‘camurupim’ was the vernacular name given to the adults of the same species. Renck et al. [15] similarly observed that ‘sauna’ was the name given to the smaller fish *Mugil curema*, while ‘tainha’ was the name given to the bigger fish. Notably, the vernacular names of some of the fish identified as uncertain within this study could represent a fish size of the target species undocumented in the literature. Another important consideration is that one vernacular name could be given to multiple scientific names. Evidence of this within my study included *Amblygaster leiogaster* and *Mugil cephalus*, named ‘kawla’ and ‘kabala’ respectively; while the spelling assigned by Suresh et al [32]. differ, the pronunciations of both fish are very similar. In another example, ‘menji’ (*Liza macrolepis*) was the vernacular name assigned to small mullets under eight inches within Chilika [39]. The same vernacular name attributed to different fish species is found in other fishing contexts; Pinto et al. [5] also reported similar findings, wherein fishermen identified *Thalassophryne nattereri* and *Batrachoides surinamensis* as ‘pacamon’.

### Gender and age observations

We found no notable differences in names assigned to fish based on gender. A lack of stark differences could be the result of males catching fish, while females are involved with the post-fishing activities related to those same fish (cleaning, cutting, cooking, or in some cases sorting the catch). Based on observations via audio recording, females sounded more confident than males in their responses; potentially because of their more intimate connection with the fish from cleaning, cutting and cooking the various species. Women can look in the eyes of the fish in the manner they deal with in comparison to men.

However, the identification of ‘bhekti’ and ‘borogo’ were exceptions to this observation about gender differences. For the most part, identification success was higher in males compared to females across age groups and villages. ‘Borogo’ and ‘bhekti’ are both high-value fish species and comprise five and one percent of commercial catch, respectively [40, 41]. A probable explanation for this nuance is that the high-value nature of these fish species might result in fish being sold to the fish traders before they even make it to the households, resulting in fewer interactions for females within these fish species. An NGO director commented,

"*high value fish they are mainly selling to the godowns [fish trader] and they are selling to the big whole sellers; they are doing export and everything only for the money they’re getting for good amount of return. And mainly middle value fish and low value fish*,* they are consuming and retaining it into the local areas*."

We found no notable differences in names assigned to fish based on age. When conducting interview surveys, we observed that seniors had a harder time recalling and identifying vernacular names. A response often heard is that they knew the fish, but could not remember their name, indicating age peculiarity. These findings paralleled that of Baird & Flaherty [37], who identified Lao fishers between the ages of 40 and 55 to be most suitable to interview because of their substantive experience, stating that senior fishers have stopped fishing and therefore their memory would be more distant. A similar finding was observed in Ayantunde et al. [24], wherein a lower number of plant species were identified by elders, likely as a result of shifting herding duties to younger men. When a ninety-year-old Naikulapatana male was asked to identify species of importance for him, a female villager responded saying,

"*if he continues to speak then he will be saying fishes which are from outside Odisha. He is not able to remember the fishes name. He’s an old man now*."

A notable observation is with regards to the fish, ‘jagili’ (*Gerres erythrourus*), which was identified as such by only four Naikulapatana fishers, all seniors. One consideration when working with local knowledge with regard to fish identification is that some species are extirpated, uncommon, or may not be contemporarily targeted within the fishing grounds [42]. One fisher commented on the differences in fish composition in the following,

"*Means the production of fish are decreasing day-by-day in the Chilika area. And most of the fish*,* also they are telling that*,* before they are getting like 200*,* 216 pieces and so they are getting more and more different types of fish. So now more and the different types of fish are not available into the Chilika area*."

### Ambiguities in scientific nomenclature

Linnean taxonomy is the universal classification approach for fish nomenclature within the scientific community [43]. The result of this approach is a description of morphological features assigned to each fish, its relationship to other fish, and a conclusive name [43]. While the scientific naming system is highly regarded within the academic community, limitations include disagreements in naming conventions between ichthyologists, naming conventions are not as accessible to non-taxonomic professionals, and revisions to taxonomic classification [43]. In regards to the last, the high value species ‘boroga’ was referred to as *Pseudosciaena coibor* by Jones & Sujansingani [39], and *Daysciaena albida* by Suresh et al. [32] in Chilika. In another example, ‘dangala’, an economically important fish species, was initially identified as *Liza macrolepis* by Jones & Sujansingani [39], *Liza troschelli* by Banerejee et al. [44], and *Planiliza macrolepis* by Suresh et al. [32] in Chilika.

In some cases, ambiguities in naming exist within both the scientific and the vernacular naming conventions. Serrao et al. [45] applied a technique called DNA barcoding for the snakehead fish (family: Channidae) to create a reference library of DNA sequences to aid in species identification. Their results concluded that *Channa striata*, *Channa marulius*, and *Channa gachua*, are cryptic species complexes, wherein each of these three “species” likely comprise multiple species and require further investigation. Differences in vernacular naming also exist across these species within the literature; *Channa striata* is identified as ‘seula’ [32] and ‘gadisha’ [35], *Channa marulius* is identified as ‘saala’ [32] and ‘chenga’ [35], and *Channa punctata* is identified as ‘gadisha’ [32] and ‘sahala’ [35].

### Implications for fish identification in Chilika Lagoon

An understanding of the linkage between vernacular and scientific naming is critical for management interventions within the context of locally important fish and keystone species. It was determined the keystone species within Chilika Lagoon to be ‘bhekti’ *(Notopterus notopterus)*,* ‘*dhala khuranti*’ (Rhabdosargus sarba)*, ‘haribolia khuranta’ (Karanteen seabream, *Crenidens creniden*), ‘verenda/udari’ (*Datnioides polota)*, ‘borogo’ *(Daysciaena albida)*, ‘fali’ *(Notopterus notopterus)*, *Chitala chitala*, ‘seula’ *(Channa striatus)*, ‘gadisha’ (*Channa punctate)*, ‘saala’ (*Channa marulius)* [46]. In considering the work by Swain et al. [46] within the context of this study, several considerations exist. As previously articulated, identification of keystone species *‘*dhala khuranti*’*, ‘verenda/udari’, ‘fali’ are geographic-dependent, indicating that these species are likely not found throughout the lagoon. Secondly, ambiguities in naming convention have previously been reported in the literature for species ‘seula’, ‘gadisha’ and ‘saala’, and warrant further taxonomic investigation [45].

The ecological keystone designation is only one component of designating a species as important; missing from this equation are the social dimensions [47]. The work by Swain et al. [46] provide a good starting point for considering key species to be protected within Chilika Lagoon. However, the ecological dimension is not enough; there is a need to consider dimensions including historical, economic, sustenance, and cultural. A dichotomy exists with species ‘borogo’ and ‘bhekti’, that creates challenges for species conservation. While their keystone designation indicates the need for their conservation to maintain ecosystem functioning [46, 48], they are also among the most commercially sought after fish species because of market demand and price [49]. This creates difficulties in conserving these two species, as they are a valuable source of income for Chilika fishers.

### Limitations and future work

This study represents a starting point in understanding patterns in vernacular naming conventions across three ecologically and geographically disparate communities within Chilika Lagoon and highlights the path forward for future work. There is a need to broaden this perspective to include a larger number of communities, as well as representation across all fisher castes and lagoon ecology. Secondly, within each species, the “uncertain” classification given to vernacular names requires further probing, as they could represent an accepted name given by the community, but undocumented in the literature. For example, the name ‘baya’ for fish *Eleutheronema tetradactylum* was originally listed as “uncertain”; however, consultation with a Berhampur fish trader revealed this to be an accepted name for smaller-size fish, despite the lack of reference within the Chilika literature. In another example, Jones & Sujansingani [39] identified *Arius arius* (written elsewhere as *Tachysurus arius*) as ‘singada’ or ‘gondia’. Within Gajapati Nagar, 11% of contributors identified this fish as either ‘gandiala’ or ‘gandila’. Further investigation is required to determine if ‘gandi[a]la’ is a variation of ‘gondia’, or an inaccurate name. Due to logistical constraints, we were unable to return to these communities to further probe our findings.

*Eleutheronema tetradactylum* (fourfinger threadfin) is given the names ‘baisiali’, ‘baiya (Sahali)’, ‘baya’, ‘baya pilla’, ‘sahala’, ‘sahali’, ‘sahalia’, ‘shahali’ within Berhampur. Inclusion of both ‘baya’ and ‘baya pilla’ suggests that fishers combine two or more criteria and/or characteristics to name a fish. For example, ‘baya’ is used as a local fish name, but some people add ‘pilla’ as a criterion to signify the size/age etc. of the fish. Further analysis or interpretation may be done to include ‘why some fishers use the term ‘pilla’ with a fish’? This may be because they simply want to refer to the size or they may be referring to the fact that a ‘pilla’ or small (child or younger) fish needs to be protected. In other words, if a ‘pilla’ fish is protected, it will be available as a bigger fish in future that will be not only economically beneficial but also produce the taste/value/quality to the fullest. This would be helpful to understand the linkage to sustainable or viable fisheries. Lastly, the most common name given across all photos was “sea fish”. This finding warrants further investigation, as this could be interpreted as an indicator of the influence of the sea on the lagoon and its communities or the intricate connections and interactions between the sea and the lagoon.

Several recommendations can be made for future studies, based on our findings. First, since photos are two-dimensional and lack information on context [25], knowledge of fish size and habitat can be provided when showing photos to the fishers. Second, because photographs might not accurately capture physical features of the fish [37], information on fish colour and scale (either provided in the photograph or mentioned verbally) can be included in the interview survey. Third, a linguist, or someone on the team that can capture vernacular names accurately in the local name, is necessary for the success of this work because of dialect and phonetic differences. Fourth, a pilot study to explore the social variables most important for capturing diversity of names (age, gender, village, etc.), can help target where sampling efforts would be most effective for capturing a diversity of names. Finally, while many studies target “expert” fishers, the consideration of a random sampling approach would allow for results to be more representative of the local community and their knowledge on fish and could provide insights into important social and ecological fish.

## Conclusions

Fisheries management and conservation requires a strong linkage between scientific research and local practices and knowledge. To do this, fishers, resource managers, and scientists require a shared language to exchange their perspectives and knowledge to develop a comprehensive understanding of fish species and to improve fisheries management. This paper set out to compare vernacular naming conventions across three ecologically and geographically disparate locations within Chilika Lagoon, India. This was achieved by showing colour photos (*n* = 56) of important fish species to local community members within three fishing villages.

Findings from this study revealed a high range of vernacular names assigned to each species, with many of these names attributed to minor phonetic differences. Second, there were no notable gender differences in fish identification except, men were able to identify several fishes with higher success than women. In situations of uneven fish distribution, accurate fish identification was higher in locations where fish were caught, demonstrating the importance of taking basin-wide approaches.

## Supplementary Information

Supplementary Material 1

Supplementary Material 2

Supplementary Material 3

Supplementary Material 4

Supplementary Material 5

## Data Availability

No datasets were generated or analysed during the current study.
