# Supplementary material for: Variations in vernacular naming of important species across three fishing villages of Chilika Lagoon, India
Source: J Ethnobiol Ethnomed. 2026 Mar 25;22:34. doi: 10.1186/s13002-026-00848-x (PMC13085592; doi:10.1186/s13002-026-00848-x)
Supplement: Supplementary file 1 — Supplementary Material 1 [file 13002_2026_848_MOESM1_ESM.docx]

**Appendix 1**: Table comparing names attributed to fish that have multiple species (*n*=12) within one vernacular name. Vernacular name indicates name assigned by village, code represents the PowerPoint slide identifier, scientific name and Odiya name were given by Suresh et al. (20), Berhampur identification/ Noikulapatana identification/ Gajipathinagar identification indicate names ascribed by villagers, and location in lagoon indicates occurrence within Chilika. Note, location in lagoon is taken directly from Suresh et al. (20).

| **Vernacular Name** | **Code, Scientific name, Oriya name** | **Berhampur Identification** | **Noikulapatana Identification** | **Gajipathinagar Identification** | **Location in Lagoon** |
| --- | --- | --- | --- | --- | --- |
| Khuranti/Khuranta | A-1 *Rhabdosargus sarba*  Dhala Khuranti | White Khuranta* | Jagili | Khuranti | Abundantly found throughout the lagoon |
|  | A-2 *Acanthopagrus longispinnis* Kala Khuranti | Black Khuranta | Khuranta | Kala Khuranti | Mainly occurs in outer channel sector and rarely found in central and southern sector of Chilika |
|  | A-3 *Acanthopagrus berda* Kala Khuranti | Black Khuranta | Khuranta | Kala Khuranti | Species mainly occurs in the outer channel and central sector of the lagoon |
|  | A-4 *Crenidens crenidens* Haribolia Khuranta | Khuranta (Brown) | Khuranti | Kala Khuranti | Smaller size sparid fish (Perch), distributed in central, southern and outer channel and rarely caught from northern sector. |
| Polagana | B-1 *Ambassis ambassis*  Bada Polagana | Polagini* | Pholla (pologona) | Pologani | Occurs predominantly in central and outer channel sector. |
|  | B-2 *Ambassis gymnocephalus*  Polagana Chandi | Polagini | Pologona | Pologani | Occurs throughout the lagoon, more abundantly in the outer channel sector. |
|  | B-3 *Chanda nama*  Bada Polagana | Brown Polagini | Sun Patia | Pologani | Catch is negligible. |
|  | B-4 *Parambassis ranga*  Polagana / Lal Chandi / Gua Chipi | Chandi; rare time in Chilika | Sea fish, no knowledge name | Pologani | Fish is distributed throughout the lagoon. |
| Jagili | C-1 *Gerres erythrourus*  *Jagili* | Jagala | Chandi | Jagala | Occurs throughout the lagoon, abundantly in central and southern sectors. |
|  | C-2 *Gerres filamentosus* *Jagili* | Jagala* | Chandi | Jagiri | Occurs throughout the lagoon and major catch comes from central and southern sectors. |
|  | C-3 *Gerres limbatus*  Jagili | Jagala | Chandi | Jagala | Mostly captured from central and southern sector of Chilika |
|  | C-4 *Gerres macracanthus*  Jagili | Kokoroba | Chandi | Jagala | Mostly found in southern sector of Chilika Lake, also occurs rarely in other sectors. |
|  | C-5 *Gerres oyena*  Jagili | Jagala | Chandi | Jagala (sea) | Occurs throughout the lagoon |
|  | C-6 *Gerres phaiya*  Jagili | Jagala | Chandi | Jagala | Fish occurs in all four sectors of the lake and forms a good commercial fishery |
|  | C-7 *Gerres setifer*  Jagili | Jagala | Chandi | Jagala | Distributed throughout the lake, more  abundantly occur in the eastern zone of  southern sector. |
| Jhudango | D-1 *Sillaginopsis panijus*  Tooldanti Jhudanga | Gaugia/Jhudango | Jhudango | Gannar | Fishery with low catch |
|  | D-2 *Sillago sihama*  Jhudanga / Kadma | Jhudango | Jhudango | Jhudango | Species is distributed throughout the lagoon. |
|  | D-3 *Sillago vincenti*  Jhudango | Jhudango | Jhudango | Jhudango | Species is distributed throughout  the lagoon. |
| Menji | E-1 *Planiliza melinopterus*  *Menji* | Soroda* | Menjia | Menji | Found throughout the lagoon. |
|  | E-2 *Planiliza subviridis*  Menji | Sorodi* | Sorada | Parsey | Fish is distributed throughout the lake but more abundant in central sector. |
|  | E-3 *Crenimugil seheli*  Magi Menji | Dhaulua | Kabala | Kabala | Fish is found throughout the lake, more abundant in central and southern sector. |
| Samadho | F-1 *Siganus canaliculatus*  Samadho / Ora | Samadho* | Potala | Ghira | Species frequently found in outer  channel area, but also found in central and southern sector of Chilika. |
|  | F-2 *Siganus javus*  Samadho/Ora | Samadho* | Potala | Ghira | Species frequently found in outer channel area, but also found in central and southern sector of Chilika. |
|  | F-3 *Siganus vermiculatus*  Samadho / Ora | Samadho* | Potala | Ghira | Fish predominantly occurs in the outer channel and also distributed in central and southern sector and also occasionally in northern sector. |
| Chauli Patua | G-1 *Corica soborna*  Ursi, Nadi Chauli | Chauli Patua | Chauli Patua | Patua | Fish occurs throughout the lagoon |
|  | G-2 *Escualosa thoracata*  Luni Chauli | Chauli Patua | Can’t say | Patua | Fish occurs throughout the lagoon. |
|  | G-3 *Stolephorus commersonnii*  Manohari Chauli Patua | Chauli Patua | Chauli Patua | Patua | Species is found throughout the lagoon. |
|  | G-4 *Stolephorus dubiosus*  Chauli Patua | Chauli Patua | Chauli Patua | Patua | Distributed throughout the lagoon |
|  | G-5 *Stolephorus indicus*  Chauli Patua / Bali Kokali | Chauli Patua | Chauli Patua | Patua | Well distributed in central, southern and outer channel sector and less in northern sector. |
|  | G-6 *Atherinomorus duodecimalis*  Lamba lunichauli | Chauli Patua | Can’t Say | Kargil | Does not form a fishery and its catch is mixed with the brackishwater miscllaneous group |
|  | G-7 *Atherinomorus lacunosus*  Samudra Chauli | Chauli Patua | Chauli Patua | Kargil | Occurs rarely |
|  | G-8 *Corica soborna*  Ursi, Nadi Chauli | Chauli Patua | Chauli Patua | Patua | Fish occurs throughout the lagoon. |
|  | G-9 *Escualosa thoracata*  Luni Chauli | Chauli Patua | Chauli Patua | Patua | Fish occurs throughout the lagoon. |
|  | G-10 *Stolephorus commersonnii*  Manohari Chauli Patua | Chauli Patua | Chauli Patua | Patua | Fish species is found throughout the lagoon. |
| Sankucha | H-1 *Narcine timlei*  Bijuli Sankucha | Sulei | Sankucho | Surei | Doesn’t really mention anything about this |
|  | H-2 *Brevitrygon imbricata*  Katia Sankucha | Sulei | Sulei | Sankucho | Occurs in the outer channel sector and occasionally near Magarmukh and Nalabana. |
|  | H-3 *Brevitrygon walga*  Dhala Sankucha, Sulei | Sulei | Sulei | Sankucho | Frequently found in the outer channel sector and in the central and southern sectors of the lagoon. |
|  | H-4 *Himantura uarnak*  Baghua Sankucha | Sankuca | ? | Chitra Sankucho | Ray fish occurs in the outer channel sector mostly during post-monsoon – summer and its rare occurrence has been observed in the central sector. |
| Kantia | I-1 *Mystus cavasius*  Bai Kantia | Singuda | Maduri Kantia | Musairi Jalango | Substantial quantity at Jaguleipadara fish landing centre under Kanasa Block and at Kalupadaghat landing centre in the northern sector. |
|  | I-2 *Mystus gulio**  Chilika Kantia | Kantia * | Luni Kantia | Kantia | Occurs throughout the lagoon. |
|  | I-3 *Mystus vittatus*  Gagar Kantia / Tengara Kantia | Kantia | Bai Kantia | Kantia | Frequently found in the northern  sector, particularly in the river mouth zone and occasionally in the central sectors  of the lake. |
|  | I-4 *Nemapteryx caelata*  Luni Kantia | Singuda | Singida | Gandiyal | The species is locally known as Luni  Kantia in Odia, caught in the outer channel mostly during post-winter and summer  when higher salinity prevails in the outer channel. |
| Danti | J-1 *Congresox talabonoides*  Danti | Danti* | Danti | Danti | Caught mostly from outer channel and central sector of Chilika. |
|  | J-2 *Muraenesox bagio*  Samudra Danti | Danti (small) | Danti | Danti | Caught from outer channel and central sector of Chilika |
|  | J-3 *Muraenesox cinereus*  Danti | Danti | Danti | Danti | Catch in Chilika is included in the mixed catch of marine eels and  all marine ells are called as Danti in Odia. |
| Chandi | K-1 *Ambassis gymnocephalus*  Polagana Chandi | Polagani | Pholla | Pologani | Occurs throughout the lagoon, more abundantly in the outer channel sector. |
|  | K-2 *Parambassis ranga*  Polagana / Lal Chandi / Gua Chipi | Pamplet | Can’t say | Pologani | Fish is distributed throughout the lagoon. |
|  | K-3 *Aurigequula fasciata*  Tankachandi | Chandi | Tonki Chandi | Chandi | Fish is regularly caught in outer channel |
|  | K-4 *Leiognathus equulus*  Tanka Chandi | *using this because it is distributed throughout the lagoon | Tonki Chandi | Chandi | Distributed throughout the lagoon. |
|  | K-5 *Nuchequula blochii*  Chandi | Chandi | Tonki Chandi | Chandi | Occurs in northern and central sector of Chilika. |
|  | K-6 *Photopectoralis bindus*  Tonki Chandi | Chandi | Tonki Chandi | Chandi | Fish is caught in association with other Leiognathid species in the outer channel sector. |
|  | K-7 *Drepane punctata*  Ghee Chandi | Pamplet | Parei | Sea Chandi | Commonly caught from outer channel sector and rarely from central sector. |
|  | K-8 *Monodactylus argenteus*  Rupali Chandi | Pamplet | Can’t say; not available | Pamplet | Rarely occurs in outer channel sector of Chilika and Palur canal. |
|  | K-9 *Monodactylus kottelati*  Chandi | Pamplet | Sea Fish | Pamplet | Rarely seen in the lake being caught in the ***Khandas*** in the outer channel and  southern sectors. |
|  | K-10 *Ephippus orbis*  Chandi | Kula Khuranti (not Chilika) | Can’t Say | Khuranta | Catch in Chilika is negligible and hence mixed with the miscellaneous brackishwater fish group. |
|  | K-11 *Scatophagus argus*  Chitra chandi | Patalia | Potala | Pathura | Occurs mainly in southern and outer channel sector and rarely in central sector. |
|  | K-12 *Acanthurus mata*  Moothia, Kala Chandi | Samadho | Can’t Say | Kau/Kokoroba | Cish has little interest to fisheries because of its small size and rare occurrence in the lake. |
|  | K-13 *Acanthurus triostegus*  Bagha Chandi | Samadho | Can’t Say | Sea Fish | Fish is occasionally found in the outer channel  and Magarmukh area in the central sector. |
|  | K-14 *Ambassis gymnocephalus*  Polagana Chandi | Pologara | Phollo | Pologani | Occurs throughout the lagoon, more abundantly in the outer channel sector. |
| Baligirida | L-1*Acentrogobius masoni*  Luni Baligirida | Bali-ghoroda | Bali-ghoda | Bali-ghoda | Occurs in central and southern sector  of Chilika. |
|  | L-2 *Drombus globiceps*  Gaji Baligirida | Bali-ghoroda | Bali-ghoda | Bali-ghoda | The fish mainly occurs in Nalabana area of  central sector to Satapada and also found in northern sector of Chilika. |
|  | L-3 *Oligolepis acutipennis*  Chhita Lanja Baligirida | Bali-ghoroda | Bali-ghoda | Bali-ghoda | Occurs mainly between Nalabana and Satapada area of Chilika. |
|  | L-4 *Psammogobius biocellatus*  Neuli Baligirida | Bali-ghoroda * | Bali-ghoda | Bali-ghoda | Occurs throughout the lagoon but frequent in central and northern sector of Chilika. |

Note: Audio of interview was not taken. Spellings of fish names for each village were written by NS (English-speaking author), and as such, do not reflect the Odiya pronunciation. Star represents fish found in abundance in for Chilika locals
