# Supplementary material for: Variations in vernacular naming of important species across three fishing villages of Chilika Lagoon, India
Source: J Ethnobiol Ethnomed. 2026 Mar 25;22:34. doi: 10.1186/s13002-026-00848-x (PMC13085592; doi:10.1186/s13002-026-00848-x)
Supplement: Supplementary file 2 — Supplementary Material 2 [file 13002_2026_848_MOESM2_ESM.docx]

**Appendix 2:** Interview survey data sheet illustrating socio-economic data collected alongside fish identifications for 56 photos

**Fish Identification Activity**

Code:_____________ Gender:_________________ Age:__________________

1. Caste:____________________

**Fish Identification**

| 1 |  | 21 |  | 41 |  |
| --- | --- | --- | --- | --- | --- |
| 2 |  | 22 |  | 42 |  |
| 3 |  | 23 |  | 43 |  |
| 4 |  | 24 |  | 44 |  |
| 5 |  | 25 |  | 45 |  |
| 6 |  | 26 |  | 46 |  |
| 7 |  | 27 |  | 47 |  |
| 8 |  | 28 |  | 48 |  |
| 9 |  | 29 |  | 49 |  |
| 10 |  | 30 |  | 50 |  |
| 11 |  | 31 |  | 21 |  |
| 12 |  | 32 |  | 52 |  |
| 13 |  | 33 |  | 53 |  |
| 14 |  | 34 |  | 54 |  |
| 15 |  | 35 |  | 55 |  |
| 16 |  | 36 |  | 56 |  |
| 17 |  | 37 |  |  |  |
| 18 |  | 38 |  |  |  |
| 19 |  | 39 |  |  |  |
| 20 |  | 40 |  |  |  |
