# Supplementary material for: Variations in vernacular naming of important species across three fishing villages of Chilika Lagoon, India
Source: J Ethnobiol Ethnomed. 2026 Mar 25;22:34. doi: 10.1186/s13002-026-00848-x (PMC13085592; doi:10.1186/s13002-026-00848-x)
Supplement: Supplementary file 3 — Supplementary Material 3 [file 13002_2026_848_MOESM3_ESM.docx]

**Appendix 3**: Table of local names assigned to each species, organized by village. Numbers accompanied by the vernacular names represent number of contributors that have assigned that particular name to the species. Each species also has the order, English common name and Odiya common name associated with it.

| **Order (photo number)** | **Scientific Name** | **English Common Name (Odiya Comon Name)** | **Distribution taken directly from Suresh et al. (2018)** | **Berhampur** | **Naikulaptana** | **Gajapati Nagar** |
| --- | --- | --- | --- | --- | --- | --- |
| Anguilliformes (27) | *Anguilla bengalensis* | Indian mottled eel (Bami) | Northern sector (sea mouth)  Occasionally in the central sector | Baligarada (1), Bomi (3), Bumi (2), Dana (1), Danti (1), Denua (1), Gomi (7), Halua (2), Kamunda (1), Kundala (1), Sapa Fish (snake) (1), Snake Fish (1), Thodi (3), Pass (11) | Danti (1), Dona (22), Dono (2), Duna (1), Gomi (3), Kenchua (1), Khuchia (1), Kunchia/Dona (1), Saula (1), Pass (3) | Bami (3), Bami/Bomi (1) Bammi (1), Bomi (9), Danti (1), Danti Pilla (1), Dona (1), Gomi (1), Jada (4), Jada (full of oil) (1), Jada (lives in a hole) (1), Jada Machha (1), Jada Pilla (1), Tudi (2), Pass (8) |
| Anguilliformes (50) | *Congresox talabonoides* | Indian pike conger (Danti) | Outer channel and central sector of Chilika | Chandi (1), Dandi (1), Danti (21), Dati (1), Gania (1), Gayala (1)**,** Gopi Snake (1), Halua (1), Samadou (1), Pass (7) | Chilika Fish (1), Dandi (2), Danti (20), Dona (2)**,** Gajia (2)**,** Gania (1),  Gomi (1), Gumi (1)**,** Pass (6) | Danti (29), Halia (1), Putura (1), Pass (5) |
| Beloniformes  (7) | *Strongylura strongylura* | Spottail needlefish (Gania) | Distributed throughout the lagoon | Gania (27), Saragada (1), Saragara (5), Pass (3), | Gania (36) | Budha Gania (4), Gania (28), Gania (Bhuda) (1), Gania (Budha) (2), Pass (1) |
| Beloniformes  (8) | *Hemiramphus far* | Black-barred halfbeak (Saragara) | Mainly found in the northern sector, followed by the central sector  Southern and central sector, occurrence is negligible | Danti (1), Gania (3), Kabala (1), Kokili (1), Nahama (1), Paniakhia (1), Saragala (1), Saragara (16), Thodi (1), Pass (10) | Gania (7), Gania Like (1), Saragada (4), Saragara (16), Sea Fish (1), Sea Fish- pass (1), Soada (1), Sorada (1), Udari (1), Pass (3) | Bada Gania (1), Budha Gania (1), Chauli Gania (2), Chauli Gania Saragara (1), Chhota Gania (1), Eka Thuntia/Chaula Gania (1), Ekathanti Gania (1), Gaanra (1), Gania (13), Gania (Kadur) (1), Gania (Mai) (1), Gania (Sarala) (1), Jhurunga (1), Jhrunga (1), Kanta Patua (1), Mai Gania (1), Paniaakhia (1), Sarbara Gania (1), Saragania/Saragara (1), Pass (4) |
| Beloniformes (9) | *Hyporhamphus limbatus* | Congaturi halfbeak (Saragara / Ekdonti) | Distributed throughout lagoon | Gania (4), Saragala (1), Saragara (25), Pass (6) | Gania (10), Sara (2), Saragada (4), Saragara (14), Sorada (1), Pass (5), | Chaula Gania (1), Gania (24), Gania (Eka Thantia) (1), Gania (Hauli) (1), Kadari Gania (1), Kadiri Gania (1), Mai Gania (1), Sabala (1), Samudra Gania (1), Saragara Gania (1), Pass (3) |
| Characiformes (55) | *Piaructus brachypomus* | (Rupchandee) |  | Bahala (1), Bhandalia (1), Kala Bainshi (1), Kala Khuranta (1), Khuranta (1), Pamplate (1), Pamplet (1), Pampplate (1), Patalia (1), Rupchandi (2), Rupchhandi (1), Samadou (2), Sea Chandi (1), Sweet water (1), Pass (20) | Abuli (1), Bada Chandi (1), Bada Jagili (1), Chandi (4), Jagili (1), Jagiri (1), Kalai Khuranta (Chandi) (1), Rupchandi (13), Rupchhandi (1), Sea Chhandi (1), Sea Potala (1), Sea Jagala (1), Udari (1), Pass (8) | Bhandaria (1), Chandi (1) Chandini (1), Gheera (1), China Kau (1), Gheera (Chaandi) (1), Gheera Chhandi (1), Glass Kau (1), Kamunda (type) (1), Khuranti (2), Madhura Macha (2), Pamplate (1), Ranguala (1), Rupchandi (6), Verindi (1), Silver Kau (1), Pass (13) |
| Clupeiformes (18) | *Tenualosa ilisha* | Hilsa shad (Ilishi) | Breeds in freshwater zone of northern sector of Chilika | Balagi (1), Balanga (10), Balangi (5), Illish (1), Ilishi (2), Illishi (4), Kanti (1), Orati (1), Paniakhia (1), Pani akhia (1), Pilli Illishi (1), Pass (8) | Balanga (1), Balingi (5), Pilla (1), Pilli (2), Pilli Illishi (1), Ilishi (1), Illishi (22), Pass (3) | Balanga (6), Balangi (3), Balingi (3), Bhakura (1), Illishi (16), Kabala (1), Kanta (1), Katha Parei (1), Kau (1), Samudra Macha (1), Pass (2) |
| Clupeiformes (30) | *Stolephorus commersonnii* | Commerson’s anchovy (Manohari Chauli Patua) | Fish species is found throughout the lagoon | Baya (1), Chaula Patua (1), Chauli (5), Chaulia (3), Chauli Patua (5), Patua (1), Sea fish (1), Sebatia (1), Serana (1), Singada (1), Sweet water (1), Pass (15) | Bada Patua (2), Chaula Patia (1), Chauli Bada Patua (1), Chauli Patia (1) Chauli Patua (6), Chauli Patua (4 types of Patua) (1), Denga Patua (1), Jaradi (1), Khati Pohala (1), Menthili (1)  Paniakhia (1), Parei- sea fish (1), Patua (4), Rahama (1), Sea fish (1), Soradi (1), Pass (11) | Bada Kukuli (1), Baunsha Patua (1), Dhobala Baligada (1), Jhurunga (1), Jalandara (long fish) (1), Kargil (1), Kokali (4), Kokoli (2), Kukoli (1), Manahari (1), Nahama (1), Patua (different) (1), Patua (2), Sahalia (1), Samudra Machha (1), Samunda Macha (1), Serana (1), Suna Kera (1), Pass (13) |
| Clupeiformes (53) | *Dussumieria elopsoides* | Slender rainbow sardine (Nadiakhai Kokoli) | Rarely caught from Chilika; only in the outer chanel sector during winter and summer | Bada Chauli (1), Bekti (1), Chaulia (1), Kawa (1) Kokoli (3), Marua (2), Nahama (4), Pani Akhia (1), Patua (3), Sea Fish (1), Sebatia (2), Sebatia Baby (1) Tirana (1), Pass (14) | Balanga (1), Chauli Patua (1), Illishi Pilla (1), Khanda Jaradi (1), Kokali (4), Kokoli (2), Marua (3), Moti (1) Parei (1), Pani Aakhia (1), Paniaakhia (1), Pilli (1), Samudra Jaradi (1), Sea Fish (2), Pass (15) | Bada Kabala (1), Kabala (3), Kabala-sea (1), Kanta Patua (1), Kila (1), Kukuli (1), Marua (4), Maura (1) Minji (1), Nahama (2), Paniaakhia (1), Samudra Fish (1), Samudra Macha (3), Samudra Machha (2) Pass (sea Fish) (1), Pass (12) |
| Clupeiformes (54) | *Stolephorus indicus* | Indian anchovy (Chauli Patua / Bali Kokali) | Well distributed in central, southern and outer channel sector  and less in northern sector. | Angarua (1), Chauli (8), Chaulia (2), Chauli Patua (4), Chuli (1), Patua (2), Sea Fish (2), Sebatia (2), Pass (14) | Bada Patua (2), Baligada Pilla (1), Chaula Patua (1), Chauli Patua (6), Chuli Patua (1), Kokali (1), Paniaakhia (1), Parei (1) Patua (5), Phasi Patua (1), Rahama (1), Saragada (1), Sea Fish (1), Sea Kokali (1), Sea Patura (1), Pass (sea fish), (1) Pass (10) | Bada Kokoli (1), Baligada (1), Baunsha Pati (1), Chauli Patua (1), Kabala (Samudra) (1), Kaanlia (soft) fish (1), Khuranti (type) (1), Kokali (6), Kokoli (3), Manohari (1), Minji (1), Paniaakhia (1), Patua (1), Sahali (1), Sahalia (1), Samudra Macha (2), Sea Fish (1), Pass (11) |
| Cypriniformes (13) | *Labeo rohita* | Indian major carp (Rohi) | The fish is frequently found in the northern  sector, particularly in the river mouth zone  in the northern sector and occasionally in  the central sectors of the lake. | Angarua (1), Bhakura (5), Kau (1), Mitikali (4), Rohi (16), Silver Cup (1), Silver Kau (2), Sweet Water (2), Pass (4) | Bhakura Pilla (1), Kala Bainshi (1), Kala Mainshi (3), Kala Mainshia (1), Mirikali (1), Mitikala (1), Mitikalia (1), Mitikaria (1), Rohi (13), Rohia (8), Rohi Chhua (1), Rohi Pilla (2), Rohia Pilla (1), Small Rohi (1), Pass (1) | Bekata (1), Bhakura (2), Glass Kau (1), Grass Cop (1), Kerandi (1), Madhura Macha (2), Madhura Machha (1), Paniakhia (1), Rohi (19), Rohia (1), Ruhi (1), Pass (3) |
| Cypriniformes (14) | *Cirrhinus reba* | Minor carp (Pohola) | Fish is a riverine migrant and thus  distributed mainly along the northern  sector of the lagoon. | Kabali (2), Khainga (1), Menjia (1), Mitikali (6), Moti (1), Rohi (5), Silver Kau (1), Soradi (1), Pass (18) | Pohada (6), Pohala (24), Kala Bainshi (1), Mitikala (1), Pass (4) | Bhakura (1), Chaanra (1), Grall Kau (1), Glass Kau (1), Kabali Pilla (1), Kabala (2), Kerandi (3), Khanda Chaanra (1), Menji (5), Menjia (1), Mira (1), Mirikali (3), Nahama (2), Paniakhia (1), Rohi (1), Ruhi type (PASS) Sea Fish (1), Surudi (1), Sweet water fish (1), Pass (7) |
| Cypriniformes (15) | *Gibelion catla* | Indian major carp (Bhakura) | River migrant, the species is caught  mainly from northern sector of the lagoon. | Bada Kala Khuranta (1), Bhakura (16), Fresh/sweet water (1), Kau (3), Khuranta (1), Khuranti (1), Kokaraba (1), Peti (1), Rohi (3), Pass (Chilika fish) (1), Pass (sweet water) (1), Pass (6) | Bada Kerandi (1), Bekati (1), Bhakura (20), Bhakur Pilla (1), Bhakura Chhua (1), Bhakuri Pilla (1), Bhakuria (2), Bhakuria (Pilla) (1), Bhala (1), Khurusia (1), Rohi (2), Rohi Pilla (1), Pass (3) | Bekata (type) (1), Bhakura (15), Bhakura Pilla (1), Boraga (1), Gilasa Kau (1), Illishi (1), Kala Khuranti (1), Kau (2), Khuranti (2), Madhura Macha (1), Mai Rohi (1), Pathara Tuka (1), Rohi (4), Pass (4) |
| Cypriniformes  (28) | *Osteobrama peninsularis* | Peninsular Osteobrama (Chalanta / Phula Kerandi) | Very rarely found in  the lagoon mainly in the northern sector. | Balang (1), Balanga (2), Paniakhia (1), Patua (1), Polagana (2), Polagara (4), Pologara (1) Polagini (7), Polagini Big (1), Sea Fish (2), Pass (14) | Bada Polagana (1), Chandi (1), Chandi (sea fish) (1), Jhala/Jahala (1), Kanagarada (1), Kanjia (2), Kerandi (1), Khuranti (1), Khurushia (1), Mahuradi (1), Patua (1), Paturi (1), Polagana (1), Polagano (1), Rabwala (1), Ranjia (1), River fish (1), Pass (Nadi Macha) (1), Pass (17) | Bada Polagana (1), Kokali (1), Not sure (Rohi/Bhakura) (1), Phhali (1), Phuluguni (2), Polagani (2), Polagini (4), Puluguni (7), Samudra Macha (1), Samundara Chandi (1), Soft Chilika Fish (1), Pass (14) |
| Cypriniformes (29) | *Systomus sarana* | Olive barb (Serena) | Fish is distributed only in northern sector  dominating the freshwater zone near the river outfall zone | Balanga (1), Bhakura (6), Bhakura Baby (1), Chhala Kerandi (1), Dhelaakhia (Bhkura) (1), Kau (1), Kerandi (5), Paniakhia (1), Rohi (5), Sea fish (1), Silver Cup (1), Silver Kau (1), Sweet Water (2), Pass (9) | Bhakura (1), Chalana (1), Chhalana (3),  Chhala Kerandi (1),  Kerandi (5), Keranti (1), Paniaakhia (1), Rohia Pilla (1), Sarana (1), Serana (18), Serena (2), Pass (1) | Bada Kerandi (1), Bekata Pilla (1), Bhakura (1), Chhala Kerandi (1), Gilasha Kau (1), Kau (1), Kerandi (16), Keraandi (Bada)/ Serena (1), Kerandi Pilla (Big Size) (1), Madhura (1), Paniakhia (1), Rohi (6), Ruhi (1), Pass (4) |
| Cypriniformes (33) | *Labeo gonius* | Kuria labeo (Khursia) | Fish occurs occasionally in the river mouth zone of northern sector during monsoon season | Bekat (Baby) (1), Bhakura Pilla (Baby) (1), Bhusa (1), Mitikali (1), Rohi (1), Rohi Bhakura (1), Sea fish (2), Seba (2), Sebatia (3) Sebatia (Seba) (1), Pass (22) | Hurishia (1), Kala Bainshi (1), Kala Khuranti (1), Khurishia (6), Khurshia (3), Khursia (2), Khurushia (4), Khurushia Pilla (1), Khurusia (4), Mitikali (1), Miti Karia (1), Rohia Pilla (1), Pass (10) | Baunsha Patari (1), Bekata Pilla (1), Bhakura (1), Bhakura Pilla (1), Grass Cop (1), Jalita (1), Magura (1), Mirikali (1), Nahama (3), Paniakhia (1), Rohi (1), Rohi Pilla (1), Sahala (1), Seba (2), Pass (19) |
| Cypriniformes  (40) | *Pethia ticto* | Firefin barb (Kuji Karandi) | Occurs in Chilika in the freshwater zone of northern sector and Nalabana area in the central sector | Angalua (1), Balangi (1), Jagala (2), Kerandi (20), Rohi (2), Soradi (1), Pass (9) | Bada Kerandi (1), Chhotta Serana (1), Chota Kerandi (1), Karandi (1), Kerandi (18), Kerandi Pilla (1), Piti Keradi (1), Serana (10), Serana Pilla (1), Pass (1) | Bekta Pilla (1), Kerandi (33), Pass (2) |
| Cypriniformes (42) | *Labeo rohita* | Indian major carp (Rohi) | The fish is frequently found in the northern  sector, particularly in the river mouth zone  in the northern sector and occasionally in the central sectors of the lake. | Balanga (1), Bhakura (4), Kau (1), Mitikali (6), Rohi (8), Rohi- Grass cup (1), Sebatia (1), Silver Kau (1), Soradi (1), Sweet Water (1), Pass (11) | Bhakuria (1), Hurushia Pilla (1), Pohada Pilla (1), Kabala (1), Kerandi (1), Mitikala (2), Mitikali (1), Mitikala Pilla (1), Mitikalia (1), Mitikalia Pilla (2), Mitikaria Pilla (1), Rohi (7), Rohia (5), Rohia Jaanla (seed) (1), Rohi Pilla (2), Rohia Pilla (2), Rohya (1), Serana (1), Serana Pilla (1), Pass (3) | Bekata Pilla (1), Dandakiri (1), Giraidi (1), Grass Kau (1), Kerandi (2), Khuranti (1), Madhura Macha (1), Madhura Machha (1), Mira (3), Mirikali (1), Mirikali Pilla (1), Mithukoli (1), Paniaakhia (1), Pani Siranga (1), Rohi (10), Rohi Jaanla (1), Rohi Pilla (1), Rohi- Grass Kau (1). Ruhi (1), Pass (5) |
| Elopiformes (5) | *Elops machnata* | Tenpounder (Nahama) | Majorly caught through gill nets from outer channel, central and  southern sectors of the lagoon | Chilika fish; freshwater (1), Kabala (1), Nahama (18), Nahamo (1), Seba (2), Sebatia (3), Sebatia (Baby) (1), Sweet Water (1), Pass (8) | Jagiri/Jagili (1), Kabala (1), Khurishia (1), Menjia (1), Nahama (1), Parei (1), Rahama (6), Rahanga (10), Paniakhia (2), Paniaakhia (4), Seba (2), Sorada (3), Pass (3) | Illishi (1), Kanta Patua (1), Nahama (25), Samudra Macha (1), Seba (2), Pass (6) |
| Gonorynchiformes  (31) | *Chanos chanos* | Milkfish (Seba Khainga) | Found throughout the lagoon | Illishi (1), Nahama (4), Parei Baby (Pilla) (1), Seba (5), Sebatia (11), Sweet water (1), Tirana (1), Pass (12) | Kala Khuranti (1), Khurushia (1), Mitikala (1), Nahanga (1), Pani akhia (1), Paniaakhia (1), Parei (1), Raanga (1), Raham (1), Rahama (1), Rahanga (3), Rahangi (1), Seba (10), Sorada (2) Sorada Pilla (1), Pass (9) | Gaara (1), Nahama (8), Nahama Pilla (1), Paniakhia (1)  Rohi (??) (Chilika) (1), Seba (11), Seba Khainga (2), Seba Pilla (1), Pass (10) |
| Mugiliformes  (10) | *Rhinomugil corsula* | Corsula mullet (Kekanda) | Occurs majorly  in northern sector between Kalupadaghat and river confluence point | Balikhaee (1), Balikhai (3), Dangili (1),  Fresh/sweet water (1),  Kabala (7),  Kabali (1),  Kabali Pilla (Baby) (1),  Kekanda (2),  Kikanda (1), Menjia (2),  River fish (Noi) (1),  Sorada (1),  Soradi (5), Pass (9) | Bhakuria (1), Dangali (1), Kabala (2), Kakenda (1), Kekanda (27), Kekaranda (1), Mengia (1), Pass (2) | (Menji)- Surudi (1), Bali Gada (4), Bali Garada (2), Bali Ghasada (1), Bali Sorada (1), Baligada (4), Balighasada (1),  Kabala (5), Kekanda (1),  Kekaranda (2), Kekaranda (Tip of my Toung) (1), Kerkenda (1), Menji (6),  Menjia (1), Soradi (2),  Surudi (1), Pass (1) |
| Mugiliformes (19) | *Planiliza macrolepis* | Largescale mullet (Dangala) | Fish occurs throughout the lagoon | Chaanri (1), Chilika fish (1), Dagili (1), Daneli (1), Dangala (1), Dangali (1), Dangeli (1), Dangili (8), Kadisa (1), Menjia (2), Sorada (12), Soradi (5), Pass (1) | Dangala (9), Dangali (2), Dangara (2), Dangeli (1), Dangili (4), Kabala (6), Mengia (1), Menji (1), Menjia (4), Mitikala (1), Sahala (1), Sorada (1), Soradi (2), Pass (1) | (Menji)-Dangala (1), Soradi Chhaanra (1), Kabala (1), Kabali (2), Mai Phasi (1), Mai Soradi (2), Menji (10), Menjia (7), Menji-Soradi (1), Minjia (1), Parshi (1), Pharshi (4), Sorada (1), Soradi (2), Pass (1) |
| Mugiliformes (26) | *Mugil cephalus* | Flathead mullet (Chilika Khainga) | Occurs throughout the lagoon | Chaari (1), Dangala (1), Dangili (2), Kabala (13), Kabala (small) (1), Kabali (5), Khainga (Khaanga) (1), Khainga (2), Menjia (3), Pathara Kata (1), Sorada (4), Soradi (2) | Dangala (1), Dangili (4), Kabala (20), Kabara (1), Mengia (1), Menjia (5), Mitikala (1), Sahala (1), Soradi (1), Pass (1) | Chaanra (1), Kabala (14), Kabala Pilla (1), Kabali (2), Kabali- (Kabala Pilla) (1), Khainga (1), Mai Soradi (1), Menji (10), Menjia (2), Pharshi (1), Samudra Machha (1), Soradi (1) |
| Mugilidae (35) | *Osteomugil cunnesius* | Longarm mullet (Soradi) | Distributed throughout  the lagoon. | Bekati (1), Chaanra (4), Chaanri (1), Chahari (1), Chanaara (1), Chanara (4), Chanra (2), Chari (1), Dangili (1), Minji  (1), Menjia (5), Patua (1), Sebatia (1), Sorada (2), Sorada Pilla (baby) (1), Soradi (8), Pass (1) | Chaanra (3), Chanaari (1), Chanra (1), Dangili Pilla (1), Kabala (4), Kabala Pilla (1), Menja (2), Menjia (16), Menji (1), Menjia Pilla (1), Sahala (1), Sorada (2), Soradi (1), Pass (1) | Aienshia Chhanara (1), Chhanar (1), Jhudunga (1), Jhurunga (1), Khanda Chaanra (1), Menji (19), Menji (one type) (1), Menji Pilla (1), Menjia (7), Minji  (1), Pass (2) |
| Mugiliformes (38) | *Planiliza melinopterus* | Otomebora mullet (Menji) | Found throughout the lagoon | Chaanra (1), Chaanri (1), Dangali (2), Dangili (7), Kabali (1), Menjia (5), Sebatia (1), Solada (1), Sorada (12), Soradi (4), Soroda (1) | Dangala (5), Dangali (2), Dangara (1), Dangili (3), Dangili Pilla (2), Dangiri (1), Kabala (5), Kabali (1), Menja (3), Menjia (8), Menjia (looks like) (1), Mitikalia (1), Sorada (2), Soradi (1) | Chhaanra (1), Dangala Pilla (1), Menji (14), Menjia (7), Minji (1), Kabala (5), Kabala Pilla (1), Kabali (1), Soradi (3), Soradi (Bada) (1), Pass (1) |
| Mugiliformes (43) | *Chelon parsia* | Goldspot mullet (Parsi Soradi) | Distributed throughout the  lagoon | Bhakura (1), Chaanra (1), Chaanri (1), Dangala (3), Dangali (1), Dangeli (1), Dangili (9), Kabali Pilla (Baby) (1), Menjia (2), Minjia (1), Sorada (11), Soradi (2), Soroda (1), Pass (1) | Dangala (6), Dangari Pilla (1), Dangili (2), Dangili Pilla (2), Kabara (1), Kabala (5), Kabala Pilla (1), Menjia (12), Sahala (1), Sorada (2), Soradi (3) | Bada Jhurunga (1), Kabala (1), Kabala Pilla (1), Kabali Pilla (1), Mai Menjia (1), Mai Pharsi (1), Menji (11), Menjia (6), Menjia (small Kabala) (1), Minji (1), Pharshi (4), Surudi (1), Soradi (5), Pass (1) |
| Mugiliformes (44) | *Osteomugil cunnesius* | Longarm mullet (Soradi) | Distributed throughout  the lagoon | Chaanra (4), Chanaara (2), Chanara (3), Dalua (1), Dangili (2), Kabali (2), Menjia (3), Minjia (1), Nahama (2), Patua (1), Sebatia (3), Sorada (2), Pass (10) | Chaanhari (1), Chaanra (4), Chhaanra (1), Dangala (1), Dangili (1), Illishi (1), Kabala (3), Kabali (1), Kabara Pilla (1), Menja (1), Menjia (4), Menjia Pilla (1), Mitikalia (1), Pania Khia Rahama (1), Paniaakhia (1), Paniakhia (1), Seba (1), Sorada (2), Pass (9) | Aamisha Menjia (1), Aienshia Chhanara (1), Amisha Menji (1), Aainsihia Kabala (1), Andra Menja (1), Chhanara (1), Jagara (1), Kabali Pilla (1), Khanda Chhanra (1), Khanda Chhara (1), Mai Pharshi (1), Menji (12), Menjia (3), Minji (1), Nahama (1), Nahama (Lots of Kanta) (1), Paniaakhia (1), Soradi (1), Pass (5) |
| Mugiliformes (48) | *Mugil cephalus* | Flathead mullet (Khainga) | Occurs throughout the lagoon | Balanga (3), Dalua (1), Dangala (1), Dangili (1), Dangli (1), Kabala (7), Kabali (3), Kakala (1), Khaienga (1), Khainga (9), Khaingia (1), Menjia (1), Sebatia (1), Sorada (1), Soradi (1), Pass (4) | Dangala (2), Dangali (1), Dangari Pilla (1), Kabala (15), Kabali (1), Kabali Pilla (1), Khaanga (1), Khaina (5), Khinga (1), Khurisia (1), Menjia (4), Pass (2) | Amisha Menji (1), Kabala (10), Kabala/Menji (type) (1), Khainga (7), Khuranti (1), Mai Menjia (3), Menji (8), Minji (1), Pass (3) |
| Myliobatiformes (23) | *Himantura uarnak* | Honeycomb stingray/Banded whiptail stingray (Baghua Sankucha) | Occurs in other channel sector mostly during post-monsoon- summer and its rare occurrence has been observed in the central sector | Chandi (1), Chitala (15), Sangucha (1), Sankucha (13), Sulei (1), Surei (1), Pass (4) | Bada Jagili (1), Kanta Machha (can't remember) (1), Magar (1), Magara (2), Pata Pata (1), Sankucha/Bahalia (Sankus) (1), Sankucha/Sulei (1),  Sankucha (14), Sulei (5), Surei (1), Pass (8) | Bada Surunga (1), Magura (1), Pichika (1), Pienki- Sankucha (1), Sankucha (20), Surei (8), Pass (4) |
| Osteoglossiformes (11) | *Notopterus notopterus* | Bronze featherback (Fali) | Generally encountered in northern and  central sectors of the lagoon | Bahalia (1), Balia (1), Dangili (1), Fali (1), Kanti (1), Pamplate (1), Pamplet (like) (1), Patua (2), Phali (3), Pompret (pamplate) (1), Rohi (1), Samadou (1), Sea Fish (4), Sea Pomfret (1), Sweet Water (1), Tirana (1), Pass (14) | Fali (1), Phali (6), Phalli (27), Phari (1), Phhari (1) | Bahalia (2), Chandi (1), Gani (1), Ghee (1), Parei/Phalli (1) Phali (3), Phalli (8), Phhali (1), Phhalli (4), Magura (1), Samundra Machha (1), Sea Fish (1), Pass (11) |
| Perciformes (2) | *Eleutheronema tetradactylum* | Fourfinger threadfin (Sahala) | Abundantly found  and well distributed throughout the  lagoon | Baisiali (1), Baiya (Sahali) (1), Baya (2), Baya Pilla (1), Bekati (1), Sahala (8), Sahali (13), Sahalia (4), Sahalia (Baby) (1), Sahalia/Baya (1), Shahali (1), Pass (2) | Bi-Sahali (1), Dangali (1), Seba (1), Sahala (18), Sahali (12), Sahalia (3) | Nahama (1), Sahalia (small) (1), Sahala (18), Sahalia (11), Shahala (1), Pass (5) |
| Perciformes (3) | *Rhabdosargus sarba* | Goldlined seabream (Dhala Khuranti) | Fish is abundantly found throughout the lagoon | Kanti (1), Khurandi (1), Khuranta (29), Khuranti (3), Pass (2) | Chandi (12), Changuna/Chandi (1), Desi Chandi (1), Dhala Jagili (1), Jagala (7), Jagali (2), Jageli (1), Jagili (7), Khuranta (1), Pass (Madhura) (1), Pass (2) | Khuranta (10), Khuranti (22), Pass (4) |
| Perciformes (6) | *Ambassis gymnocephalus* | Bald glassy (Polagana Chandi) | Occurs  throughout the lagoon, more abundantly in the outer channel sector | Polagana (1), Polagani (1), Polagara (6), Polagini (19), Polanga (1), Rohi-Bhakura (1), Pass (7) | Bada Polagana (1), Chandi (1), Gua Chipi/Polagandui (1), Pada Polagana (1), Phulla (1), Phulla (Sun Patia) (1), Polagana (19), Polagara (2), Sun Patia (2), Sunu Patia (2), Sunu Patua (1), Pass (looks like Chandi) (1), Pass (3) | Kokali (1), Phuluguni (4), Polagana (1), Polagani (7), Polagania (1), Polagini (1), Pologani (1), Puluguni (11), Puluguni (Bada) (1), Tirana (1), Pass (7) |
| Perciformes (12) | *Datnioides polota* | Silver tiger perch (Verenda / Udari) | Occurs throughout the lagoon but  more frequently found in central and northern sector | Angalua (1), Angalua (Black) (1),  Angarua (8),  Angarua (Baby) (1),  Bekti (Baby) (1),  Dhinkia (2),  Kala Bainshi (1),  Kala Khuranta (2),  Kau (1),  Khuranta (Black) (1),  Kokaraba (1),  Kundala (2),  Kundali (2),  Kundalia (2),  Pathara Takua (1),  Pathara Thakua(1),  Samadou (1), Pass (7) | Chandi (1), Jagili (1), Kala Khuranta (1), Khuranta (8), Khuranti (1), Udali (1), Udari (20), Udari-Kala Khuranti (1), Rohi (1), Pass (1) | Kala Bahala (1), Kala Khuranti (2), Kau Puri (Full of Bones) (1), Kerandi (1),  Khuranti (1), Khuranti (type) (1), Kokaraba (1), Kundala (1), Pathamundi (stonehead) (1), Pathar Thuga (1), Pathara Mundi (1), Pathara Thuka (3),  Pathara Tuga (1), Pathara Tugutuka (1), Pathara Tuka (6), Pathara Tukutukua (1), Pathara Tunka (1), Patharatuka (1), Ranguala (1), Thunta Kundala (1), Verandi (2), Verendi (1) |
| Perciformes (16) | *Channa striata* | Striped snakehead (Seula) | Frequently found in the northern  sector, particularly in the northern part of northern sector and occasionally  in the central and southern sectors of the lake. | Balikhai (1), Bekta (1), Gadisha (5), Kau (1), Saula (13), Sea fish (1), Seula (5), Silver Kau (1), Pass (8) | Desi Saula (1), Gadisha (3), Gadisha (Phulia) (1), Saula (26), Saulia (1), Seula (3), Pass (1) | Gadisha (7), Saula (2), Sehula (1), Seula (23), Shaula (1), Shahala (1), Pass (1) |
| Perciformes (17) | *Lates calcarifer* | Barramundi / Asian seabass (Bhekti) | Distributed throughout the lagoon | Bekati (16), Bekta (5), Bekti (5), Black Khurant (1), Chagala (1), Mitikali (Bekti) (1), Pass (7) | Bekata (5), Bekati (4), Bekati Pilla (1), Beketa (1), Bekiti (1), Bekta (8), Bekta (King of Chilika) (1), Bekta (big) Chagali (small) (1), Bekti (5), Lamba Udari (2), Khuranta (1), Khuranti (1), Pass (5) | Bekata (9), Beketi (1), Beketa (2), Bekiti (2), Bekta (7), Bekti (2), Bhakura (1), Bhekti (2), Pathara Tuka (1), Pass (9) |
| Perciformes (20) | *Etroplus suratensis* | Pearlspot (Kundala) | Occurs throughout  the lagoon | Kala Khuranta (2), Kundal (1), Kundali (2), Kundalia (4), Kunadala (1), Kundala (21), Samadou (1), Pass (4) | Chandi (1), Desi Khuranta (1), Jagili (1), Khuranta (2), Khuranti (1), Kundala (26), Kundla (1), Mundi Udari (1), Pass (2) | Pathara Thuka (1), Kundala (34), Pass (1) |
| Perciformes (25) | *Daysciaena albida* | Bengal corvine (Borogo) | Occurs throughout the lake | Bekati (1), Bekti Baby (Pilla) (1), Bhakura (1), Black Khuranta (1), Boraga (14), Boragi (1), Boroga (1), Borogo (2), Kokara (1), Kokaraba (6), Pass (7) | Bekati (1), Bekati Pilla (1), Boraba (1), Borag Kania (1), Boraga (20), Boraga Kania (3), Boragakani (1), Boragali (Boraga) (1), Borog-Khania (1), Boroga (1), Khuranti (1), Kania (1), Pass (3) | Bekati (1), Boraga (14), Boroga (2), Borogo (1), Buroga (2), Burogo (2), Burugo (4), Khuranti (1), Pathara Mundi (1), Pilla Bekata (1), Pass (7) |
| Perciformes (32) | *Pomadasys argenteus* | Silver grunt (Kokoraba) | Occurs throughout the lagoon but more frequent in central  and northern sector than other two. | Angarua (1), Boroga(1), Kala Khuranta (2), Khuranta (8), Khuranti (1), Kokarab (1), Kokaraba (19), Kokoraba (1), Pass (2) | Bekati (2), Bhunga Boroga (1), Boraga Kania (1), Boraga type (1), Boraga (1), Chandi (8), Ghee Chandi (2), Jagala Khuranta (1), Jagali (2), Jagili (3), Jagiri (1), Kala Jagiri (1), Kala Khuranti (1), Khuranta (2), Khuranti Pilla (1), Kokali (1), Udari (1), Udari Chhua (1), Pass (5) | Bada Khuranti (1), Jagiri (4), Kala Bekti (1), Kala Khuranta (1), Kala Khuranti (1), Khuranta (2), Khuranti (10), Khuranti (type) (1), Kokaraba (3), Pathara Mundi (3), Pathara Thuka (2), Pathara Tuka (1), Verendi (1), Verindi (1), Pass (4) |
| Perciformes (34) | *Pomadasys kaakan* | Javelin grunter (Kokoraba) | Occurs throughout the lagoon but  more frequent in central and outer channel sector. | Bhakura (1), Boraga (Baby) (1), Boraga (3), Khuranta (2), Kokaraba (24), Pass (5) | Bekati Pilla (1), Boraga (1), Boraga Kania (1), Chandi (4), Dhala Chandi (1), Ghee Chandi (1), Gojia Jagili (1), Goouguan (1), Gunguan (1), Jagili (2) Jagiri (1), Kala Khuranti (1), Khuranta (3), Sankara (sea fish) (1), Sea Fish (3), Pass (13) | Bada Khuranti (1), Bekti (1), Khuranta (type) (1), Boraga (1), Borogo (1), Burogo (1), Burugo (1), Burugu (Boraga) (1), Jagiri (6), Khuranti (3), Khuranta (3), Kokaraba (3), Kukura Khia Khuranta (1), Kukuruba (1), Pathara Mundi (1), Pathara Tuka (1), Samundar Fish (1), Pass (8) |
| Perciformes (36) | *Gerres filamentosus* | Whipfin silver-biddy (Jagili) | Occurs throughout the lagoon and major catch comes from central  and southern sectors | Jagala (24), Jagali (1), Jagiri (1), Kala Khuranta (2), Kau (1), Khuranta (1), Patharakata (1), Pass (5) | Bada Jagili (1), Bekati Pilla (2), Chandi (17), Dhala Chandi (1), Ghee Chandi (2), Jagela Chandi (1), Jagili (1), Jagiri (1), Kala Ghee Chandi (1), Kala Khuranta (1), Khuranta (1), Pass (6), Khuranti (1) | Jagala (6), Jageri (1), Jagili (2), Jagiri (18), Kala Khuranta (1), Khuranta (1), Kau (1), Khuranti (3), Pass (3) |
| Perciformes (37) | *Sillago sihama* | Silver sillago (Jhudanga / Kadama) | Fish species is distributed throughout  the lagoon. | Gajia (14), Jhodanga (3), Judunga (1), Jhudanga (6), Jhudunga (2), Sea Fish (1), Soradi (1), Pass (8) | Baligada (4), Jhudanga (12), Jhudunga (1), Rahama (1), Sea Fish (2), Sorada (2), Soradi (2), Suun (1), Pass (11) | Bali Sorunga (1), Baligada (2), Jhudanaga (1), Jhudanga (4), Jhudunga (10), Jhurunga (4), Jhurunga (canal) (1), Kukuli/Kokali (1), Suranga (2) Surunga (2), Surunga/Jhudunga (1), Surungi (1), Pass (6) |
| Perciformes (39) | *Siganus javus* | White-spotted spinefoot (Samadho/Ora) | Species frequently found in outer channel area,  but also found in central and southern sector of Chilika | Khuranta (1), Samadau (1), Samadho (1), Samadou (25), Samodou (3), Samudou (3), Pass (2) | Chandi (4), Jagili (1), Jagili Pilla (1), Jagiri (1), Kau Khia (1), Khuranta (2), Khuranti (2), Kou/Kau (1), Paniaakhia (1), Parei (3), Potala (9), Potola (1), Rupchandi (1), Saland (1), Samudra Potala (1), Pass (5), Sea Fish (1) | Putura (1), Ranguala (1), Bada Gheeri (1), Ghee (1), Geera (1), Gheera (13), Gheera Pilla (1), Ghera (1), Ghera Chandi (1), Ghira (1), Gira (4), Gira/Ghira (1), Khuranti (2), Sea Fish (1), Pass (6) |
| Perciformes (41) | *Scatophagus argus* | Spotted scat (Chitra chandi) | Fish occurs mainly in southern and outer channel sector and rarely in central  sector. | Patalia (29), Samadou (1), Pass (6) | Bhandari Khura (1), Bharei (1), Chandi (1), Gaana (1), Jagili (1), Khuranti (1), Potala (13), Potola (2), Sea Chhandi Pilla (1), Sea Fish (1), Tanki Chandi (1), Pass (12) | Chandi (1), Chhandi (2), Ghee (Variety) (1), Gheera (2), Ghira (1), Gira (1), Ptura (1), Putura (22), Pass (5) |
| Perciformes (45) | *Carangoides ferdau* | Blue trevally (Parei) | N/A | Kadisha (1), Kantei (1), Kanti (12), Kokaraba (1), Samadou (1), Languda Moda (sea fish) (1), Sea Fish (2), Tirana (12), Pass (5) | Chandi (3), Gaana (2), Ghee Chandi (1), Jagili (1), Kanagarda (1), Karkabara (1), Kathisa (1), Kathisha (3), Kathisia (1), Kokali (1), Languda Moda (1), Lanja Moda/Languda Moda (1), Para (1), Parei (1), Potala (1), Potali (1), Samudra Potala (1), Sea Chandi (1), Sea Fish (3), Pass (10) | Bada Tirana (1), Chandi (1), Gheea Chhandi (1), Kanti (2), Katha Parei (1), Kathi Parei (1), Kathisha (1), Kokaraba (1), Parei (1), Samudra Chhandi (1), Tirana (8), Tirina (1), Sea Fish (1), Pass (15) |
| Perciformes (46) | *Terapon jarbua* | Jarbua terapon (Gahana) | Well distributed  throughout the lake but abundant in south-east part of the lake | Gaana (35) Pass (1) | Angalua (1), Boraga Kani (1), Chandi Boraga (1), Gaanna (1), Gaana (12), Gahana (3), Khuranti (1), Luni Kau (1), Potala (1) Rahanga (1), Sea Fish (2), Pass (11) | Gaana (1), Gandiala (1), Munda Gaana (25),  Munda Gaahana (1),  Muda Gahana (1), Tadigiri (2), Tarigiri (1), Pass (4) |
| Perciformes (51) | *Leiognathus equulus* | Common ponyfish (Tanka Chandi) | Distributed throughout the lagoon | Balikhai (1), Bhandalia (1), Chandi (29), Chandini (1), Khuranta (1), Paniakhia (1), Pass (2) | Chandi (21), Chhandi (2), Ghee Chandi (1), Gunchandi (1), Jagala (1), Jagili (1), Jagiri (1), Tankei Chandi (1), Tanki Chandi (2), White Chandi (1), Pass (4) | Baligada (1), Chandi (15), Chhandi (15), Ghia Chandi (1), Jagiri (1), Sea Chandi (1), Pass (2) |
| Perciformes (52) | *Psammogobius biocellatus* | Sleepy goby (Neuli Baligirida) | Occurs throughout the lagoon but frequent in central and  northern sector of Chilika | Bali Garada (1), Bali Khai (2), Baligarada (6),  Baligaradi (1), Balikhaee (2), Balikhai (13), Gadisha (1), Kamunda (1), Saula (3), Sea fish (1), Pass (5) | Bali Gada (8), Baligada (23), Baligarada (1), Desi Baligada (1), Saula (2), Pass (1) | Bali Gada (5), Bali Garada (2), Bali Ghasada (1), Bali Ghosada (1), Bali Ghurusa (1), Bali Kada (1), Baligada (14), Baligada Pilla (1), Baligada/Bali Suruda (1), Baligadrada (1), Baligarada (1), Baligha Sada (2), Ghadisha (1), Menji (1), Pass (3) |
| Pleuronectiformes (22) | *Cynoglossus puncticeps* | Speckled tongue sole (Aswa) | Occurs mostly in central (eastern Nalabana area)  and outer channel sector of Chilika. | Balikhaee (1), Patua (10), Rasa Patua (6), Sea Fish (1), Thodi (1), Pass (17) | Baligarada (1), Kaanda (Kamunda) (1), Pata Pata (16), Patapata (8), Patpata (1), Patua (1), Samudra Patua (1), Pass (7) | Bhandaria (General Fish) (1), Dudhi Patua (1), Kanta Patua Patua (18), (1) Phhali (1), Pichika (1), Rasa Patua (1), Surunga (1), Thakara (1), Tudi (2), Pass (8) |
| Siluriformes (4) | *Arius arius* | Threadfin sea catfish (Singada) | Occurs throughout the lagoon | Baya (small Sahala) (1), Bekt (baby) (1), Singada (31), Sunga (Singada) (1), PASS (2) | Singada (32), Sunga (1), Suunga (3) | Gandiala (8), Gandila (3), Kantia (1), Mushiari (4), Singada (2), Singida (13), Singira (1), Sunga (1), Sungha (1), Sunguda (1), Suunga (1) |
| Siluriformes (21) | *Plotosus canius* | Grey eel-catfish (Kaunda) | Distributed throughout  the lagoon | Bhakura (1), Dolphin (1), Kamuda (2), Kamunda (22), Kumunda (1), Siengi (1), Singada (1), Singhi (3), Singi (2), Thodi (1), Pass (1) | Attaa (1), Kaanda (12), Kaaunda (1), Kamunda (8), Kaunda (5), Magura (3), Nali Kamunda (1), Rata (4), Singada (1) | Kamunda (28), Magura (5), Patua (1) Pass (2) |
| Siluriformes (24) | *Wallago attu* | Wallago / Freshwater shark (Balia) | Mostly caught in the freshwater zone of northern sector of the lake,  particularly near the river mouth zones. It is also encountered in the central sector. | Angarua (1), Bachha (1), Bahalia (1), Balia (6), Fali/Phali (1), Fani (1), Puin Singada (1), Sea Fish (2), Singada (3), Singhi (1), Singi (1), Suunga (1), Pass (sweet) (1), Pass (15) | Bahalia (11), Balia (17), Bahalia Pata (1), Bahlia (5), Kanda Balia (1), Pass (1) | Baya (1), Bahalia (11), Balia (6), Bhakura (1), Jalanga (1), Madhur Machha (1), Madura Macha (1), Magura (1), Musiari (1), Phhali (1), Singida (1), Singira (1), Pass (8), Singada (1) |
| Siluriformes (47) | *Pangasius pangasius* | Pangas catfish (Jalanga) | Occurs  only in northern sector, particularly the northern part of the northern sector. | Bacha (1), Baccha (1), Bachha (4), Bachha Pilla (1), Balia (2), Chaanra (1), Dangili (1), Kantia (1), Puan Singada (1), Singada (14), Singhi (1), Suunga (2), Pass (6) | Bacha (1), Bachara (2), Bachhara (1), Jageli (1), Jalanga (18), Jalanga Pilla (1), Maduara Singada (1), Paniaakhia (1), Sea fish/Pass (1), Singada (2), Singada Pilla (1), Suunga (2), Pass (4) | Bahalia (1), Gandiala (7), Jalanga (2), Kantia (3), Menji (1), Mushiari (3), Musiari (1), Singada (4), Singida (5), Suunga (2), Pass (7) |
| Siluriformes (49) | *Mystus gulio* | Long whiskers catfish (Chilika Kantia) | Occurs  throughout the lagoon | Kantia (18), Sweet water (1), Singada (10), Singhitali (1), Singitali (1), Pass (5) | Kanta (1), Kantia (23),  Luni Kantia (2), Singada Pilla (1), Singada (5), Singhitali (1), Singitali (1), Singitali- (Kantia/Singada) (1), Pass (1) | Gandiala (1), Kantia (29), Mushiari (2), Musiari (1), Singida (1), Suunga (1), Pass (1) |
| Siluriformes (56) | *Clarias magur* | Walking catfish (Magura) | Frequently found in the northern sector of the lake, | Kabala (1), Kamunda (7), Magura (4), Rata (3), Rohi (1), Siengi (1), Singhi (8), Singi (4), Singitali (1), Tudi (1), Pass (5) | Balanga (1), Kaunda (1), Magura (20), Rata (11), Ratta (1), Singada (1), Pass (1) | Bhakura (1), Bhakura Pilla (1), Kamuda (1), Kamund (1), Kamunda (3), Kamunda Pilla (1), Kantia (1), Magura (19), Magura (three bones) (1), Magura Pilla (1), Maguru (1), Tudi (1), Pass (4) |
| Tetraodontiformes (1) | *Tricanthus biaculeatus* | Short-nosed tripodfish (Sukura) | It is a  commonly occuring and resident species in Chilika | Chandi (3), Kanti (1), Sakura (2), Sukura (29), Pass (1) | Sukuda (8), Sukuda (Sukura) (1), Sukura (12), Sukuta (13), Pass (2) | Sukura (34), Sakura (2) |
