# Supplementary material for: Variations in vernacular naming of important species across three fishing villages of Chilika Lagoon, India
Source: J Ethnobiol Ethnomed. 2026 Mar 25;22:34. doi: 10.1186/s13002-026-00848-x (PMC13085592; doi:10.1186/s13002-026-00848-x)
Supplement: Supplementary file 4 — Supplementary Material 4 [file 13002_2026_848_MOESM4_ESM.docx]

**Appendix 4:** Bar graphs representing range of responses contained for each photograph shown to local community numbers, with the number in the bracket representing the order that the photo was shown. The local name is listed before the brackets, while the scientific name is listed within the brackets. Names listed across the y-axis represent the various names assigned by local community members, with (A) signifying accepted response, (NR) signifying not recognized, and (U) signifying uncertain. The x-axis represents number of respondents.

[1] **Sukura (*Tricanthus biaculeatus*)**

[2] **Sahala (*Eleutheronema tetradactylum*)**

[3] **Dhala Khuranti (*Rhabdosargus sarba*)**

[4] **Singada (*Arius arius*)**

[5] **Nahama (*Elops machnata*)**

[6] **Ambassis gymnocephalus (*Polagana Chandi*)**

[7] **Gania (*Strongylura strongylura*)**

[8] **Saragara (*Hemiramphus far*)**

[9] **Saragara/Ekdonti (*Hyporhamphus limbatus*)**

[10] **Kekanda (*Rhinomugil corsula*)**

[11] **Fali (*Notopterus notopterus*)**

[12] **Verenda / Udari (*Datnioides polota*)**

[13] **Rohi (*Labeo rohita*)**

[14] **Pohola (*Cirrhinus reba*)**

[15] **Bhakura (*Gibelion catla*)**

[16] **Seula (*Channa striata*)**

[17] **Bhekti (*Lates calcarifer*)**

[18] **Ilishi (*Tenualosa ilisha*)**

[19] **Dangala (*Planiliza macrolepis*)**

[20] **Kundala (Etroplus suratensis)**

[21] **Kaunda (*Plotosus canius*)**

[22] **Aswa (*Cynoglossus puncticeps*)**

[23] **Baghua Sankucha (*Himantura uarnak*)**

[24] **Balia (*Wallago attu*)**

[25] **Borogo (*Daysciaena albida*)**

[26] **Chilika Khainga (*Mugil cephalus*)**

[27] **Bami (*Anguilla bengalensis*)**

[28] **Chalanta / Phula Kerandi (*Osteobrama peninsularis*)**

[29] **Serena (*Systomus sarana*)**

[30] **Manohari Chauli Patua (*Stolephorus commersonnii*)**

[31] **Seba Khainga (*Chanos chanos*)**

[32] **Kokoraba (*Pomadasys argenteus*)**

[33] **Khursia (*Labeo gonius*)**

[34] **Kokoraba (*Pomadasys kaakan*)**

[35] **Soradi (*Osteomugil cunnesius*)**

[36] **Jagili (*Gerres filamentosus*)**

[37] **Jhudanga / Kadama (*Sillago sihama*)**

[38] **Menji (*Planiliza melinopterus*)**

[39] **Samadho/Ora (*Siganus javus*)**

[40] **Kuji Karandi (*Pethia ticto*)**

[41] **Chitra chandi (*Scatophagus argus*)**

[42] **Rohi (*Labeo rohita*)**

[43] **Parsi Soradi (*Chelon parsia*)**

[44] **Soradi (*Osteomugil cunnesius*)**

[45] **Parei (*Caranjoides ferdu*)**

[46] **Gahana (*Terapon jarbua*)**

[47] **Jalanga (*Pangasius pangasius*)**

[48] **Khainga (*Mugil cephalus*)**

[49] **Chilika Kantia (*Mystus gulio*)**

[50] **Danti (*Congresox talabonoides*)**

[51] **Tanka Chandi (*Leiognathus equulus*)**

[52] **Neuli Baligirida (*Psammogobius biocellatus*)**

[53] **Nadiakhai Kokoli (*Dussumieria elopsoides*)**

[54] **Chauli Patua / Bali Kokali** (***Stolephorus indicus*)**

[55] **Roopchandi (*Piaructus brachypomus*)**

[56] **Magura (*Clarias magur*)**
