# Supplementary material for: Variations in vernacular naming of important species across three fishing villages of Chilika Lagoon, India
Source: J Ethnobiol Ethnomed. 2026 Mar 25;22:34. doi: 10.1186/s13002-026-00848-x (PMC13085592; doi:10.1186/s13002-026-00848-x)
Supplement: Supplementary file 5 — Supplementary Material 5 [file 13002_2026_848_MOESM5_ESM.docx]

**Supplementary file 1**: List of common names in Fishbase assigned to each species listed in this study. Common names are not listed in entirety; please refer to the Fishbase page via the stock code for a full account of the names

| **Scientific name** | **English name (Suresh et al. 2018)** | **Common name (Fishbase)** | **Fishbase Stock code** |
| --- | --- | --- | --- |
| *Ambassis gymnocephalus* | Bald glassy | Ambache, Ambache du large, Ambasse, Aringil, Bald glassy, Bald glassy perchlet, Chandee, Commersons Glasbarsch, Cá Sơn biển đầu trần, Dodosi, Glassy perchlet, Glatzkopf, Got kattila, Kaalkop-glasvis, Kaljulasiahven, Kyaung-ma-cer, Langarai, Langaray, Langstekel-glasvis, Longspine glassy, Marie Jeanne, Mullu-tharu, Naked-head glassy perchlet, Nanthan, Nga-zin-cert, Pangkah, Pengkat, Pridin, Seriding botak, Seriding putih, Sorgan | 5035 |
| *Anguilla bengalensis* | Indian mottled eel | Aarel, Aerel, African mottled eel, Aheer, Ahir, Anguia, Anguila, Angula, Badung, Banehara, Batung, Belut, Belut Benggala, Bengali angerjas, Butul, Con chinh, European eel, Freshwater eel, Gal arndha, Ganga arndha, Gnalu, Hanchu menu, Hanchumeenu, Harimeenu, Igat, Indian longfin eel, Indian mottled eel, Indian mottled eel, Indian Mottled Eel, Indian mottled eel, Indisk broget ål, Kabara arndha, Kaha arndha, Longfin freshwater eel, Malamgulu, Malugu, Maniangal, Mlangil, Moorish eel, Mottled Eel, Naprum, Nga-mee-toung, Nkunga, Pol mal andha, Polon arndha, Porivelangu, Porivilangu, Pulli arndha, Raj bam, Rajbam, Rem, Roluo, Seram pambu, Serampamboo, Sinsilud, Starry moray, Thumbi, Tuna, Vali arndha, Vallangoo, Vellangoo, Vilangu, Vilangu, Z'amab, Z'amab, Z'anguille, Z'anguille, Úhoř indický | 1289 |
| *Arius arius* | Threadfin sea catfish | Bagre filamentoso, Catfish, Cá úc, Duri, Duri filament, Hamilton's catfish, Jella, Kanduli, Lundu, Marine catfish, Mâchoiron fouet, Pulutan, Siimamerimonni, Threadfin sea catfish, Trådfinnet havmalle, Utek | 1302 |
| *Carangoides ferdau*  (Synonym for *Ferdauia ferdau*) | Blue trevally | Ampahan, Apahan, Banded trevally, Bar jack, Barred jack, Barred trevally, Bayad, Belo-belo, Blou koningvis, Blue kingfish, Blue trevally, Blå trevalle, Carangue la nation, Carangue rayée, Carangue tachetée, Cupak, Cá Khế chấm vàng, Cá Khế féc-đô, Dalupani, Damis lawin, Dchtep, Demudok, Demudok biru, Fanihandi, Feraau's cavalla, Ferdau's trevally, Ferdy, Forskal's jackfish, Hamam, Istah putih mangalih, Jack, Jash, Jemudok, Jurel manchado, Kemurok, Kolekole, Kuluvel, Kurohira-aji, Lambiyan, Langog, Lawihan, Lemuduk, Lison, Mamsa, Manganinao, Mara hobu, Marapini, Nanyô-kaiwari, Nyankawe, Nyubba biasa, Omuri, Pahuru, Pakeva, Pampano, Pampanung riyal, Popo, Puharehare, Putih, Raerave, Rambai, Rewa, Sall, Sarish-chep, Scad, Shepel sarish, Shiiraan, Sibo, Simba one nduru, Talakitok, Talapion, Tarakite, Tarakito, Tarakitok, Tarokotokan, Tarotokan, Tarukitok, Tawa-ay, Tayang-tayang, Te kona, Te pula, Trakito, Ulua, Uru'ati, Urua, Urupigh, Vilu, Xaréu azul, Yellow spotted trevally | 2117 |
| *Channa striata* | Striped snakehead | Aluan, Amanu, Anak dalag, Aruan, Aruwan, Badau, Bado, Bajong, Bako, Bakuli, Bale balong, Bale salo, Banded snakehead, Bayong, Bulig, Bundaki, Bundalag, Cabeza de serpiente cabrio, Chevron snakehead, Chotiyan, Cá lòc, Cá Ló, cá Sộp, Cá lót (lóc), Cá quả, Cá Sộp, Cá träu, Dalag, Dalak, Dekhu, Delak, Deluk, Dhoali, Dok, Dolla, Gabus, Gapuran, Guelue-guelue, Haal, Haluan, Haroan, Haruan, Haruting, Helae, I'a pâkç, Kapuran, Karuppu veral, Kaunan, Koochinamari, Korra matta, Korramenu, Kuchheu, Kuchhu, Kutuk, Lawag, Loolla, Mangsha, Morl, Morrul, Mudfish, Murrel, Nga-yan, Nga-yau-auk, Obog, Pa kaw, Pa kho, Pba kaw, Phtok, Pla chon, Pongee, Pooli-kuchi, Ptuok, Quergestreifter Schlangenkopf, Raitakäärmeenpää, Randig Ormhuvudfisk, Ros, Ruan, Saura, Shawl, Shoal, Shol, Snakehead mudfish, Snakehead murrel, Sohr, Sola, Soura, Sowl, Sowrah, Stribet slangehovedfisk, Striped snakehead, Sulbud, Taki, Talosog, Telak, Terebog, Toman, Toman paya, Torabó, Trey phtuok, Trey ras, Trey raws, Trey ros (or ras), Tête de serpent strié, Utan, Viral wrahl, Vraal, Żmijogłów pręgowany | 357 |
| *Chanos chanos* | Milkfish | Aifa, Aol, Auta, Ava, Ava uta, Avali'i, Awa, Awa 'aua, Awa kalamoho, Awa-awa, Awátai, Ba'neawa, Bagas, Bagus, Balayongan, Banato, Bandang, Bandeng, Bandong, Bangilis, Banglis, Banglos, Banglot, Banglus, Bangos, Bangris, Bangros, Bangrus, Bangus, Bangyos, Banlus, Belanak sembawa, Bolu, Bony salmon, Buetil, Bugi, Bukkariba, Bunji, Buruna, Buto bangolos, Buulga, Cefalone, Chanidé, Chano, Chanos, Chanos stříbřitý, Chaol, Chaol kereker, Cá Máng, Cá Măng biển, Fiamandry, Garongin, Giant herring, Hakuna moana, Hoo-meenu, Humeen, Hyder's fish, Ikan be'e, Ilweyn, Jangas, Kaaco, Kaayo, Kakawag, Kawag-kawag, Khameh Mahi, Khanos, Kodenec, Lisa montañera, Lubine, Lumulokso, Maitokala, Mata momoivao, Matafa, Mekkunu, Melkfisk, Melkvis, Mesekelat, Miikwa, Milchfisch, Milkfish, Mjölkfisk, Mlečnica, Modbod, Moles, Moreton Bay salmon, Mwariho, Mwatiko, Mælkefisk, Ndrongo, Nemer, Nu-haok, Nu-hyaok, Oaa, Oma'a, Omaa, Pa' a pa' a, Pal-kendai, Palai-meen, Pallabontha, Palmeen, Pati, Peite-leite, Pelio, Phuane, Phwade, Piimkala, Pisang-pisang, Pla dok mai, Pla nuan chan tha la, Pla nuan chan tha-lay, Pla sa lin, Pla thu nam jeud, Poisson lait, Polah-bontah, Poomeen, Pua awa, Sabahii, Sabalo, Sabalote, Salmani, Salmaun, Salmon herring, Sandry, Sheem, Sábalo, Tamano, Te awa, Te awatai, Te bukimanai, Te'efō, Thon, Thullu, Trey nuan chan, Tulu-candul, Tulucandul, Vaikkaya, Vango, Waawa, White mullet, Yauta, Yawa, Ågua, | 95 |
| *Chelon parsia*  Synonym of (*Planiliza parsia*) | Goldspot mullet | Avele-meen, Bata, Boi, Bonthalu, Gold spot mullet, Ka-be-lu, Kanbo, Lisa mejilla dorada, Madavai, Mulet joue d'or, Nga-kin-nyin, Parshe bata, Sherto | 4885 |
| *Cirrhinus reba* | Minor carp | Arju, Aruzu, Ballalamosa, Beli krap, Cauvery white carp, Cá Mrigan, Dodda-arja, Dudu kendai, Intianmutakarppi, Jhilke, Mariga, Mirga, Mirgali, Mirigal, Mirikali, Mirki, Mirrga, Mirrgah, Mirya, Morahkee, Morakha, Mori, Movakha, Mrigal, Mrigal carp, Mrigala, Mrigalam, Nagari, Nain, Nainee, Naini, Narain, Nga-gyin, Ostroretka čtyřvousá, Pa nang chan, Pudu kendai, Trey krawlang, Trôi trắng, Valge tsirriin, Vellaranjan, Vencandi, Venkata, Venkendai, White carp, Yerramosa, Yerramosu | 55318 |
| *Clarias magur* | Walking catfish | No common name listed | N/A |
| *Congresox talabonoides* | Indian pike conger | Belut, Common eel, Conger eel, Conger-pike eel, Cá Lạt, Daggertooth pike-conger, India purikangerjas, Indian conger pike eel, Indian pike conger, Indian pike conger, Indian pike-conger, Indian putyekanipa, Indisk geddemuræne, Indong, Kamila, Ketingkor, Malong, Malong India, Morenocio indio, Morénésoce indien, Mun shin, Nong, Pambumeen, Pamuréna indonéská, Tuna, Vam, Wam | 12039 |
| *Cynoglossus puncticeps* | Speckled tongue sole | Ararali, Blotched tongue-sole, Changchun, Cá Bơn vằn, Dali dali, Dapa, Dapang sinilas, Dapang tsinelas, Iat-ilat, Kulampira, Kulampira-bato, Langue tachetée, Lempa, Lemper, Lengue moteada, Lep, Lidah, Lila, Lisan, Lisan bagar, Malad-palad, Mottled tonguesole, Pad-pad, Pahot, Palad, Speckled tongue sole, Tonguefish, Trey andat chhke | 5036 |
| *Datnioides polota* | Silver tiger perch | Barred tigerfish, Cá huòng, Firbåndet tigerfisk, Four-banded tigerfish, Four-banded triple-tail, Four-barred tigerfish, Four-barred-tiger-fish, Hõbedane vöötkolmsaba, Kyauk-nga-wet, Pla kapong lai, Trey khlar, Vielgestreifter Tigerfisch, Vierbinden-Dreischwanzbarsch | 14366 |
| *Daysciaena albida* | Bengal corvine | Bengal corvina, Bengal corvine, Bhola, Bola, Croaker, Drum, Gelama, Gelama Benggala, Goli, Gorasa, Karoom-kattelee, Kathalai, Kora, Pallikora, Panna, Pullipanna, Two-bearded croaker, Vanakathalai, Vella-kattelle, Vellaikathalai | 12375 |
| *Dussumieria elopsoides* | Slender rainbow sardine | Balatiyong, Cá Lầm bụng tròn, Daslo, Feemaru, Gin-iwashi, Hasselt's sprat, Hasselt's sprat, Hilos-hilos, Hint sardalyası, Hoi ho, Janggul, Jepuh, Kanasi, Kasig-kasig, Kolachi, Kolakoyan, Kolu baige, Lambiyawan, Madha kandai, Mennethe, Morava, Poondivirinjan, Rainbow round herring, Rainbow sardine, Round herring, Sale ümarkõhtheeringas, Sardina arco iris grácil, Sardine arc-en-ciel gracile, Sardinha, Sardinka elopsovitá, Sardinya, Sharp-nosed sprat, Siriande, Slank regnbuesardin, Slender rainbow sardine, Slender sardine, Sprat, Tamban, Tamban bulat, Tamban buluh, Tamban jepuh, Tamban-bines, Tamban-buluh bulat, Tembang bines, Teng hoon, Thondon, Tulis, Um, Van Hasselt's sprat | 1647 |
| *Eleutheronema tetradactylum* | Fourfinger threadfin | Baameen, Bahmin, Baling, Bameen, Bamin, Banakon, Barbudo de cuatro dedos, Barbudo del Indo-Pacífico, Barbudo mamalí, Barbudo-do-Indo-Pacífico, Barbure mamalí, Barbure à quatre doigts, Bastard mullets, Battihiravalai, Benong, Bikau, Blind tassel-fish, Blue salmon, Blue threadfin, Bluenose salmon, Blunt-nosed salmon, Boddumaga, Boka dulse, Budathamaga, Buka-dulce, Bukadolse, Bungot, Burnett salmon, Chetyrekhpalyi pal'tseper, Colonial salmon, Cooktown salmon, Cá chét, Cá Nhụ bốn râu, Cá Nhụ lớn, Dhalbadyi, Four finger thread fin, Four tasselfish, Four thread tassel fish, Fyrstrålig Trådfisk, Genohong, Giant tasselfish, Giant threadfin, Gubal, Guchhia, Horse's friend, Hugao, Hugau, Ikan salangan, Ilou mugoru, Indian salmon, Indo-pacifisk trådfinnefisk, Jenohong, Jubal senohong, Kagau, Kakuyan, Kala, Kalawa, Kalemeen, Kattikalai, Kesumbang, King fish, Kingfish, Kondiangalai, Kubal, Kugao, Kugaw, Kurau, Kurau janggut, Kurau pipih, Kuru janggut, Lakkha, Lausan, Lelaoh tanah, Lelauh tanda, Maga, Mamale, Mamali, Mancong, Menangin, Minami-konoshiro, Ne-nal-ga-ji, Nelikiir-sõrmkala, Nga-let-kwa, Nga-ta-yar, Norakudiyan, Panigala, Podikimaga, Pozhakkala, Pugao, Ramsi, Rashgoo maamooli, Rawas, Riesenfederflosser, Rockhampton kingfish, Rockhampton salmon, Row ball, Sahal, Sahala, Seenakalai, Selanghi, Senangin, Senangin rambu empat, Senohong, Sheem, Sheim, Sinanghi, Sinanghi/Selanghi, Soon hong, Sumbal, Sì zhĭ mǎ yǒu, Tabarara, Tailla, Tassel fish, Thamuthi, Thellamaga, Thiravalai, Thread fin, Tigi, Tigi', Trey pream, Vahmeenu, Valanakala, Vameenu, Vellaikala, Wahmeen, White salmon, Wiciak wielki, Yevakala, Štiriprsta nitkarica | 354 |
| *Elops machnata* | Tenpounder | A shin poke, Alho, Australian giant herring, Awa-awa, Banana fish, Banang bulat, Bandang, Bayirbir, Bedbed, Bid-bid, Bidbid, Big-eye giant herring, Big-eye herring, Birbir, Bony fish, Chirya, Cá Cháo biển, Dandeli, Elops indooceánský, Elops indyjski, Fateixa, Giant herring, Grande écaille, Guinée saumon, Hanisi, Hanithi, Ilweyn, Indo-Pacific tarpon, Indopatsiifiline elops, Jallagu, Jinnagow, Kihanisi, Lady fish, Lingolingo, Lubine male, Lulong, Malacho salmon, Manna, Mannava, Menangin, Mkizi, Moanjy, Mooran kendai, Mtsumbwi, Naum, Numera, Pincushion fish, Poomeen, Ranava, River skipjack, Shonas, Springer, Tangiron, Tarpon, Tempounder, Tenpounder, Tipunder, Ua, Ullahti, Vallipoomeen, Whole sardine | 5801 |
| *Etroplus suratensis* | Pearlspot | Banded etroplus, Banded pearlspot, Cashi-mara, Cashimara, Cundahle, Diamantcichlide, Duvvenachepa, Elimeenu, Erumeen, Etroplus, Green chromide, Indischer Streifenbuntbarsch, Intiankirjoahven, Irimeen, Kalunder, Karassar, Karimeen, Koraliya, Palincha, Panishettai, Pearl spot, Pearlspot cichlid, Pitul, Sethakandai, Seththa kendai, Sri Lanka kirikala, Striped chromide, Udupatti, Zóltaczek cejlonski | 354 |
| *Gerres filamentosus* | Whipfin silver-biddy | Abat like, Amolok, Amorok, Badha, Badha riashy, Bansa, Batuan, Batuhanon, Batwanon, Baysa, Bedaha, Blanche fil, Chaa, Chaghook-e-reshtehdar, Charbit, Cá móm, Cá Móm gai dài, Dokmakkradong, Filamentous mojarra, Flagfin mojarra, Geres nitkopletwy, Hamorok, Holebaige, Hubagah, Itohikisagi, Jaggari, Jagili, Kapas, Kapas besar, Kapas filamen, Kapas laut, Kapas-kapas, Kekapas, Kodianpurachi, Kotigo, Kotikou, Kotto, Lamurok, Lamuruk, Latab, Liyemwit, Long-rayed mojarra, Long-rayed silver biddy, Lumong, Majorras, Malabon, Malacapas may lawi, Malagapas, Manabon, Melanúria filamentosa, Mojarra, Mojarra de hebra, Mussalakh, Nga-se-ooh, Nga-si-ooe, Niituim-hõbeahven, Omoket, Oodaan, Oodan, Pengarat, Pranjin, Putian, Putih mata, Putihan, Rayasha, Rayasheh, Sakalan, Sala, Samin-samin, Samok, Samolok, Sedakang, Senohong, Senolong, Silver-biddy, Sweepvin-plooibek, Taboh, Tak chanda, Tampok-tampok, Threadfin pursemouth, Threadfin silver belly, Threadfin silver-biddy, Threadfin silverbelly, Trey doh Angkor, Tsarasaina, Udagam, Ulu watu, Uniyamas, Whip fin silver biddy, Whipfin mojarra, Whipfin silver biddy, Yatab, Yeegany | 4661 |
| *Gibelion catla*  (Common name of *Labeo catla*) | Indian major carp | Bacha, Bahkur, Barkur, Baudhekera, Baudhekra, Bawas, Bhakua, Bhakur, Boassa, Bocha, Botcha, Botchee, Catal, Catla, Catla catla, Catlabarbe, Chepti, Hiidkarp, Japan kendai, Kanavi, Kapr indický, Karakatla, Katal, Katala, Katalin bangla, Katla, Katla obecná, Katlya, Katol, Koora kendai, Koora-Kendai, Krishnabotcha, Major carp, Meen, Nga gaung pwa, Nga-thaing, Pla kaho tad, Pla kra ho, Tambra, Taylee, Thaila, Thambra, Theil, Theila, Theppu meenu, Thoppa, Thoppa-meen, Vakur, Yamaneri kendai | 4637 |
| *Hemiramphus far* | Black-barred halfbeak | Abo-meshfa, Abou H'arbeh, Abu-minkar, Agujeta, Agujete manchada, Agulha, Agulha peixe pica, Aiguillette, Antseradava, Antseraka, Antseraka-vonina, Baguing babae, Balanban, Balang, Balao, Balitos, Balitot, Balobo kuning, Baritos, Baritos na balang, Barred garfish, Barred halfbeak, Bayanban, Bayangban, Black barred garfish, Black-barred halfbeak, Buging, Buging babae, Bugiw, Buguin, Buloy, Bungug, Burog, Buroy, Burukun, Busa, Båndplettet halvnæb, Chuchunge, Chuchunge tili, Chuchungi, Coka-ilo, Cá Kìm chấm, Demi-bec, Demi-bec bagnard, Demi-bec à barres noires, Demi-bec à taches noires, Fela, Gaka, Gefleckter Halbschnäbler, Gevlekde halfbek, Halbschnäbler, Half beak, Halfbeak, Hnok-thido, Hoshi-zayori, I'usila, Ihe fota, Jenjulung, Jolong selang tanda, Jolong-jolong, Jung jung, Kacang-kacang, Kansusuwit, Kidau, Kuku, Laniw, Leu sula, Maming, Maminy, Meia-agulha manchada, Meming, Menyulong, Mezzobecco, Misoramfos, Muigri, Mural, Mususa, Mwâtèè, Nga-taung-myin, Nkuil n'tsantra, Nyenyulung, Pakanye, Pilangan, Polukljuna iglica, Poluryl, Puput, Puput banang, Puput bandang, Puput banding, Qaraare, Sa-sa, Salasa, Salawasid, Saltón, Sasa, Sawasid, Sayasa, Sembeh, Sembir, Senjolong, Sils, Sindik, Siriw, Sise, Spotted halfbeak, Suasid, Sulwi, Sundoprono, Sunduk, Susay, Suwasid, Swasid, Talos, Taruda nguhu, Thovulu, Todak pendek, Tracas, Tsera-Pohe, Tserapano, Tuging ubod-ubod, Täplänokkakala, Xaata, Yarımgaga balığı, Zaraganya | 5663 |
| *Himantura uarnak* | Honeycomb stingray/Banded whiptail stingray | Banded whiptail stingray, Belya, Bhatya, Bitoonan, Boor, Bouchée, Budang, Burá alveolado, Bâsternâk Hindyeh, Chupare oval, Coachwhip ray, Cá Đuối bồng gai, Dahunan, Heuningkoek-pylstert, Honeycomb, Honeycomb ray, Honeycomb stingray, Honeycomb whipray, Hyomon-otome ei, Imantoura, Indo-Australischer Tüpfelrochen, Katongganon, Kilkigan, Krabane Lai-mang-won, Lakhman, Leik-kyauk-hua-maungdo, Leopard ray, Leopard stingray, Leopard-Stechrochen, Longtail ray, Longtail stingray, Lontail ray, Luipaardpijlstaartrog, Lukhma, Lukhmah, Madi, Manal thirukai, Marbled sting ray, Marbled stingray, N'tasera, Ndrasera, Netmønstret piskerokke, Ogoncza arnak, Pagi, Paging bulik, Paging dahunan, Paging sulatan, Pangladan, Pari, Pari batik, Pari beting, Pari bunga, Pari bunga/batik, Pari kelawar, Pari macan, Pari merica, Pari pasir, Pari rimau, Pari tanjung, Pastenague indienne, Pastenague leopard, Pastenague nid d'abeille, Pilisan, Pow-e-palangy, Puli-tenke, Pulli-thirukkai, Raie alvéolée, Raie blanche, Reticulate whip ray, Ruget, Sankush, Shafane shabeellow, Shankar, Sona-kah-tirike, Tarabla, Thirandi, Trnucha hedvábná, Tırpana balığı, Uarnak-nuutrai, Uzun kuyruklu rina, Vali maduva, Waghya-pakat, Warkhol Patari, Whaghya pakat, Whiptail ray, Whiptail stingray, Yidi | 5796 |
| *Hyporhamphus limbatus* | Congaturi halfbeak | Agujeta congaturí, Congaturi halfbeak, Cá Kìm bên, Demi-bec congsturi, Garfish, Gongaturi, Halfbeak, Jolong birai hitam, Jolong-jolong, Kawaka bachcha, Khempakdaeng, Kolaan, Langgai, Marandha, Moralla, Phtinh, Puput, Sarbara, Sembir, Sils, Sumb, Surali-kandai, Todak, Todak pendek, Toli, Trey phtaung, Trey phtinh, Trey phtoung, Valenciennes halfbeak | 8627 |
| *Labeo gonius* | Kuria labeo | Assaminlabeo, Ghainna, Ghonia, Gonya, Gurdi, Karsa, Khursa, Kuri, Kuria labeo, Kuria-narmasmokk, Kursa, Labeo ganžské, Minor carp, Nga-dein, Shada ghonia | 10577 |
| *Labeo rohita* | Indian major carp | Bhobhari, Bocha-gandumeenu, Cá Trôi, Cá Trôi Ân Độ, Cá Trôi Ân Độ, Rôhu, Debeloustni krap, Dhambra, Dum-bra, Dumra, Jättilabeo, Kannadi kendai, Labeo avanské, Labeo rohita, Labeo Roho, Labéo Roho, Nga myait chin, Pla yee sok tad, Pla yee sok tead, Rahu, Rau, Rohhu-narmasmokk, Rohi, Rohitham, Rohiti, Rohitii, Roho labeo, Rohu, Rokhu, Row, Ruee, Ruhu, Rui, Tambada masa, Tambada massa, Tapra, Роху | 97 |
| *Lates calcarifer* | Barramundi / Asian seabass | Akame, Apaap, Apahap, Apap, Asian seabass, Balga, Baramundi, Barlga, Barra, Barramunda, Barramundi, Barramundi perch, Barrumundi, Bekkut, Bekti, Bhekti, Birloonkoordany, Bolgan, Brochet de mer, Bukai-bukai, Bulgan, Bulungan, Burgan, Cabeh, Cabik, Cock-up, Cock-up seaperch, Cockup, Cukil, Cá Chẽm, Cá vurot, Cá Vược, Dadhara, Dayang, Deyo, Durruah, Fitadar, Gariyu, Gelungsung, Giant perch, Giant sea perch, Jiteda, Ka ka tit, Ka-ka-dit, Ka-ka-htit, Ka-kadit, Kaka, Kakap, Kakap puteh, Kakap putih, Kanja, Katuyot, Keduwa, Keliji, Khajura, Khajuri, Kim bak lor, Koduva, Koliji, Kulapu, Lates, Laya, Maan cho, Mangagat, Mata kuting, Matakating, Matang pusa, Modha, Murrabal, Murrulpal, Nair-meen, Nari-meen, Narimeen, Nga-tha-dite, Nuddee-meen, Painnee-meen, Palmer, Pandu-kopah, Pandu-menu, Pelak, Perca gigante, Perche barramundi, Petehan, Pica-pica, Pletekan, Poyo, Riesenbarsch, Salungsungan, Sapan, Sea bass, Seaperch, Selungsung, Siakap, Siakap putih, Silver barramundi, Silver perch, Silver seaperch, Solong-solong, Telah, Tetahan, Trey spong, Tul-wan, White sea bass | 360 |
| *Leiognathus equulus* | Common ponyfish | Almindelig ponyfisk, Anketraketra, Asurini, Badyang, Bakagan, Barurog, Bebete, Bete-bete debe door, Betebete, Cajao, Cebe, Chap-chap, Civar, Common ponyfish, Common slipmouth, Cá Liệt lớn, Dagoldalan, Daguldolan, Dalupane, Dalupani, Danutan, Dexena, Dodok, Gedabang, Greater ponyfish, Gumabek, Hiwas, Hobu-hõbekõht, Hotu panna, Huwaling, I-im, Kaikai, Kalama, Kapotol, Kedabang, Kekek, Kekek gedabang, Kekek jalur, Ketraketra, Keyam, Kikeh, Kokoko, Ladintavia, Lalakasen, Laway, Laway-laway, Lawayakan, Lawayan, Lawihan, Lumo-an, Lumoan, Maladia, Malaway, Mas karalla, Mawalay, Motambo común, Mumu, Mutomot, Mydliczek ekwula, Narrow-banded ponyfish, Nepis-nepis, Nga-dingar, Nga-waing, Orange fin pony fish, Pa-ut-put, Paenyak, Palangan, Palotpot, Paluput, Panjzari-e-bozorg, Parutpot, Parutput, Patana comum, Peperek topang, Pepetek, Pirak-pirak, Pony fish, Pulot-pulot (Bajau), Rayasha 'aridha, Sap-sap, Sap-sap betsang, Sapsap, Sapsap commun, Seitaka-hiiragi, Sekiki, Shortnose pony, Sipesipa, Slimy, Slimy soapy, Slipmouth, Slymvis, Soapy, Sooro-koo-nam-kare, Tabiros, Tak chanda, Taksay, Talibukno, Talibuno, Tambon, Tambong, Tanka-chandee, Tariptip, Tooth-pony, Trey sambow hear, Usub, Yapyap | 4649 |
| *Mugil cephalus* | Flathead mullet | Aalder, Aarder, Aguas, Agwas, Ain, Aitam, Albur, Aligasin, Ama'ama, Anace, Anae, Andapong, Anding, Antafa, Antendro, Anubah, Araaby, Asfatiya, Asubi, Auaree, Avrita balığı, Babaš, Balanak, Baldigare, Banak, Bayanak, Belanak, Beyah, Bhomat, Biah, Biah Srpehn, Biyah, Black mullet, Black true mullet, Blaneut, Boi, Boita, Bol, Bolpina, Bora, Bouchakfa, Bouri, Bouri aftess, Bouri kabir elras, Bright mullet, Bully, Bully mullet, Bullûa, Bái yǎn, Caanood, Cabezudo, Cabeçut, Cabot, Cachamba, Cagarraz, Callifaver mullet, Calmou, Cambiro, Cap pla, Capazzone, Capiton, Capitán, Capocefalo, Capozzo, Capuozzo, Carida, Carido, Caridou, Carmou, Cefalo, Cefalo Mazzone, Cefalo Verace, Cefalo vero, Cefalu, Cefolo, Cefulu cirini, Cephalos, Chefal, Chefal mare, Ciavarini, Ciefl, Cievollo, Cievolo, Cipal, Cipal batas, Cipal bataš, Cipal glavas, Cipal glavaš, Cipli, Common grey mullet, Common mullet, Cremole, Cumraku, Cumri, Curemà, Curimã, Curimã-í, Cá Đối mục, Céfalo, Deem, Diabaï, Diklipharder, Dème, Eguisse, Eirigo-do-rio, Firzetta, Flachkopf-Meeräsche, Flathead grey mullet, Flathead mullet, Galupe, Gandhia, Gawafa, Gefalu, Gemeine Meeräsche, Gereh, Gerita, Gerpuh, Gestreifte Meeräsche, Gewöhnliche Meeräsche, Gewöhnliche Meeräsche, Gharyb, Gis, Gisaw, Glavati cipelj, Glavaš, Glissà, Gray mullet, Grey mullet, Grossköpfige Meeräsche, Großkopf, Großkopf-Meeräsche, Grå multe, Guiss, Gushtaku, Gutarana, Guéseou, Haarder, Harder, Hardgut mullet, Hardgut river mullet, Harilik kefala, Has kefal, Haskefal, Haskefal balığı, Hurong, Ilissa lobarrera, Iliça de cap gros, Iliça sabada, Is barri godeya, Jabaay, Jagede, Javra, Jempol, Jompo, Jumpul, Juovakeltti, Kafal, Kahaha, Kanae, Kanahe, Kapae, Kapiiyut, Kaplat, Kasmeen, Kathi-parenga, Kathiparega, Kedera, Kefal, Kefal balığı, Kefalos, Keffal, Kifol gedol harosh, Kifon gdol hazosh, Kitheya, Koklan balığı, Koto, Kunungui, Képhalos, Kôrômii, Kûrûmil, Laban, Laiguan-asut, Lebranche, Liguan, Lisa, Lisa cabezuda, Lisa común, Lisa común, Lisa pardete, Lisa rabo Amarillo, Lisa rayada, Lisas, Lissa amaria, Liza cabezona, Lizarra, Lizza, Liça, Llissa llobarrera, Loban, Lombbie, Longaram mullet, Lul-luran, M'Hizi, Ma sek, Maalan, Machu, Machuto, Madavai, Mala, Manalei, Mangan, Mangrove mullet, Manla, Massun, Mattarello, Mazzardu, Mazzone, Mecia, Meciatino, Meciato, Mesciarino, Meuil, Miil-budhi-dhirr, Mile, Milé, Mkizi, Mollit, Morski cefal, Mpafa, Muge, Muge cabot, Muge céphale, Muge à groose tête, Mugella, Mugem, Muggine, Muggine caparello, Muggini, Mugil, Mugil australijski, Mugil cefal, Mugil común, Mugo fungous, Mujelle, Mujol, Mujou, Mule, Mulet, Mulet bleu, Mulet cabot, Mulet jaune, Mulet jeune, Mulet voile, Mulet à grosse tête, Mulet-cabot, Mulett, Mulett ta' l'iswed, Muletta, Mulettu, Mulj, Mullet, Multe, Multe, Musai, Musao neigro, Muxo, Muza, Muzao, Muzzeru, Muzzulu, Müsaro, Naxoc, Ngefanu, O'ola, Ociangne, Olhal, Olhalvo, Pahaha, Papalvo, Pardete, Perong, Pilas, Pisciammano, Platkop-harder, Poddies, Poddy mullet, Poisson queue bleue, Pordete, Porong, Pua, Pua 'ama, Pua 'ama'ama, Pua po'ola, Qefulli i veres, Qefulli i verës, Rapang, River mullet, Roumediabou, Röndungur, Sand mullet, Sea mullet, Sherto, Si-agne, Sibo, Siegolo, Sievolo, Sinal, Skakavac, Skocac, Skočac glavas, Skočac glavas, Springer, Storhovedet multe, Storhuvad multe, Stribet multe, Striped mullet, Sung-ǒ, Tagana, Tainha, Tainha cabeça achatada, Tainha-olhalvo, Tainhota, Talilong, Tamaratana, Tapiara, Tapuji, Testard, Testone, Testu, Thel godeya, Thirutha, Tiruta, Tirutha, Tistuni, Tjockläppad multe, Topan balığı, Topan kefal, Topbaş balığı, Tororaka, Tshulwa, Tueppe, Tóu zī, Ugapang, Urichoa, Utuutu, Volpina, Volpino, Vopina, Waloya, Wu tau, Wu tau tze, Wutsuma, Yellow-tailed mullet, Zievalo, Zompona, Zoulé, Zī, Zī yú | 801 |
| *Mystus gulio* | Chilika Kantia | Anguluwa, Catfish, Chinkada, Cá chot trang, Cá chuóc, Cá Chôt, Getting, Gigi, I-kong, Irung-kelutti, Kadal-kelithi, Kala-tenguah, Katta-keluthi, Keting, Kontia, Langskægget pigmalle, Long whiskers catfish, Lundu, Mang korn, Nai-keluthi, Nga-zin, Nuna-tengra, Pla e-kong, Pla kayeng noo, Pla mang korn, Pla yeng noo, Sengati, Sengkiran, Shingati, Uppang-kelettee, Uppangkeletee, Vaaleajuovamonni, Vella-koorai | 5376 |
| *Notopterus notopterus* | Bronze featherback | Almindelig knivbladfisk, Ambattan valai, Ambattan vazhai, Ambattan-kathi, Ambattankathi, Asiatic knifefish, Asiatischer Fähnchen-Messerfisch, Battu, Belida, Bronze featherback, But, Chalat, Chamari, Chappali, Chappali-chamari, Chappathi, Chennavalai, Chinna vazhai, Chital, Chottavalai, Common knife fish, Cotta vazhai, Cá thát lát, Dtong, Falui, Feather back, Foley, Foli, Golhai, Grey featherback, Harilik sulgselgkala, Harmaateräkala, Kandla, Kanduli, Kapirat, Lepsi, Moh, Nga-pe, Nožovec africký, Nožovec malý, Nožovec obecný, Pa tong, Pa tong na, Patara, Patre, Payi, Pba dtawng noi, Pholi, Pholui, Phouli, Pla cha lat, Pla cha-lad, Pla chalat, Pla sa lat, Pla sa teo, Pla sa tue, Pla sa-lad, Pla salat, Pla tong, Pla tong na, Pronssiteräkala, Purri, Slat, Spinoper, Trey slat, Ulakathatta, Vala | 8301 |
| *Osteomugil cunnesius* | Longarm mullet | Anding, Avelemeen, Balanak, Banak, Belanak, Belanak anding, Belanak bakau, Belanak kedera, Belanak kodok, Belanak putih, Bentek, Bhadvi, Boi, Buntis, Caanood, Chia hu, Cá döi, Cá dúi, Cá Đối đầu nhọn, Dhuraay-dhuraay, Gereh, Gereh kodok, Gisaw, Jempol, Kadar, Kedera, Kedera sayap Panjang, Kemurak, Kunnesee, Langarm-harder, Langfinnet multe, Lisa de aleta larga, Lisas, Loban, Long-finned mullet, Longarm mullet, Longfin grey mullet, Longfin mullet, Longfin-mullet, Lul-luran, Maid, Mkizi, Mud mullet, Muge, Mulet, Mulet longue aile, Mullet, Nan-yô-bora, Round-head mullet, Round-headed mullet, Roundhead mullet, Tainha de barbatana grande, Talilong, Thac, Wedge snout mullet, Yaanit, Yanit | 4921 |
| *Osteobrama peninsularis* | Peninsular Osteobrama | Kambalgi, Pattakunji, Peninsular osteobrama | 13514 |
| *Pangasius pangasius* | Pangas catfish | Aie, Banka-jella, Choluva-jella, Choluvajella, Comboo-keletti, Coola kelettee, Coola-kellette, Cá ba sa, Cá xanh ky, Djambal, Djuara, Eye keletee, Gulhalet hajmalle, Intianhaimonni, Jalkapoor, Jambal, Jellum, Juara, Kovailoolakeluthai, Lawang, Manga-keluthi, Pa souei, Pangas, Pangas catfish, Pangash, Pangra, Pangsa, Pangus, Pariasi, Patasi, Patin, Payas, Periasi, Pla ai duang, Pla sa wai, Pla sa wai kluay, Pla sang ka ward, Pla sang ka ward khao, Pla sang ka ward kluay, Pla sang ka wart, Pla sang ka wart khaow, Pla sang ka wart kluay, Ponga, Pongas, Ponnga, Punagas, Pungas, Pungwas, River catfish, Schwarzflossen-Haiwels, Trey pra, Vitki som, Yellowtail catfish | 306 |
| *Pethia ticto* | Firefin barb | Aurinkobarbi, Brzanka birmanska a. dwuplama, Chenaputhi, Darahi, Fire-fin barb, Kadum kalee, Kadungali, Kahanee potia, Kakslaik-pethia, Kali bein, Kaoli, Kotree, Kudgi-kerundi, Manimajra, Naya-paisa, Ngakha, Paral, Parigi, Parmička dvouskvrnná, Parmička indická, Parmička pihovatá, Parmička tečkovaná, Parmička žlutoskvrnná, Pothia, Poti, Potina, Pulli kenda, Pulli kendai, Putter-perlee, Sidre, Tetputi, Thith pethiya, Thunnus, Tic-tac-toe barb, Ticto barb, Tit punti, Tite Pothi, Titputi, Tituputi, Topletbarbe, Toplettet Barbe, Two spotted barb, Two-spot barb, Vennatii, Zweifleckbarbe, Zweipunktbarbe | 5044 |
| *Piaructus brachypomus* | Rupchandee | Cachama, Cachama blanca, Caranha, Gamitana-Scheibensalmler, Morocoto, Paco, Pacu, Pacú, Paku, Pirapatinga, Punapaku, Riesenpacu, Sølvpacu, Tambaqui, Yae cho nga moat | 6104 |
| *Planiliza macrolepis* | Dangala | Aguas, Anubah, Araby, Asae, Asfatiya, Avelemeen, Avulimeen, Balanak, Banak, Belanak besar, Belanak sisik besar, Berneo-mullet, Beyah, Biah sefeti, Big scale liza, Bingyi, Biyah, Bonthaparigi, Borneo mullet, Bui leka, Caanood, Cheluvakandai, Chiryakandai, Cá Đối vảy to, Dingliah, Gawafa, Gejameenakendai, Gisaw, Grey Mullet, Grootskub-harder, Gusaw, Gutarana, Gyalwe, Hararan, Kabalu, Kampango, Kana rath godeya, Kanambu, Kanbo, Khôlap, Kiri godeya, Ko-bora, Koniga, Kônuu, Kôrômii, Kûrûmîî, Large scale mullet, Lisa godeya, Lisas, Liza wielkoluska, Loban, Lul-luran, Madathale, Madavai, Madavakandai, Majni, Mallan, Manalai, Mkizi, Mulet lunette, Mulet rond, Mulet à grandes écailles, Mullet, Panu godeya, Pare, Polosataya kefal', Surada, Sydafrikansk storskællet multe, Tainha godé, Talilong, Thirutha, Vali godeya, Velisa, Yabi, Yaiuw, Yaiuwach, Yaiuwetang, Yayúw, Zompona | 5045 |
| *Planiliza melinopterus*  (Common name of  *Planiliza melinoptera*) | Menji | Aligasin, Araran, Balanak, Banak, Belanak, Belanak perak, Blackfin mullet, Caanood, Cream mullet, Cá Đối bạc, Giantscale mullet, Mullet, Gisaw, Godeya, Gusaw, Iokedch, Kanace, Kedera, Lisa otomebora, Lisas, Lul-luran, Manalei, Mkizi, Mulet otomebora, Mullet, Otomebora mullet, Reuseskub-harder, Saint Lucia Mullet, St. Lucia-harder, Tainha Lucia, Tainha otomébora, Talilong | 5948 |
| *Plotosus canius* | Grey eel-catfish | Anito, Bakih, Balibot canin, Barbel-eel catfish, Black-tip catfish, Canine catfish-eel, Cá ngát, Cá Ngát nanh, Daniw, Duzy sumik koralowy, Eel catfish, Gang magur, Gemang, Gray eel catfish, Hamilton's eel catfish, Heto-heto bukay, Hito, I-ito, Irung-keletee, Irung-kellettee, Ito, Kalapu magura, Kamda, Kan-magur, Kana magura, Keduthal, Kelara, Mushu, Nalshingali, Nga-nu, Oitu, Pa duk taleh, Pa lai fai fa, Pa-di, Patuna, Patuna canina, Pin-lae-nga-khu, Pla duk tale, Sembilang, Sembilang gemang, Semibilang, Semilang batu, Semilang karang, Senangin, Striped catfish eel, Thor sart, Trey andaing tonlay, Trey andeng koi, Ungilayi, Unsat, Unsat-unsat, Vari-choongum, Varichundan mushi, Wal shingala, Walshingala, Walshingti | 8478 |
| *Pomadasys argenteus* | Silver grunt | Abo, Ago-ot, Agoot, Agot, Aguot, Aguut, Akuhut, Alibalay, Ambah, Andopeng (Bajau), Angera-Bato, Bakoko, Bakoko agurt, Balay, Baraw-baraw, Besechaml, Brunplettet sølvgryntefisk, Bwâlap, Celak mata, Chelek mata, Cheri, Corocoro plateado, Cá Sạo, Cá Sạo bạc, Fine-spotted grunter-bream, Gerut-gerut, Gerut-gerut ambah, Gerut-gerut perak, Ghorakan, Gorakka, Grondeur argenté, Grondeur argenté, Grounder argenté, Grunter, Grunter bream, Head grunt, Head grunter, Hoshi-Mizoisaki, Ibalay, Ikan kepala batu, Ikuran, Iri bateya, Kadichani, Kakan, Kalianthalai, Karkara, Karukaruppan, Karuppi, Kepala batu, Khokho, Kiskisan, Kithalu vulayen, Komkee, Kurukuruppan, Kurukuruppan, Kurumutti, Kwe-kwe, Lekpeh, Lepe, Likti, Lined silver grunt, Lined silver grunt, Lined silver grunter, Luszcz hasta, Mekedchelewel, Mizoisaki, Mullankra, Nagroor, Nagrur, Naqrur, Ompak, Oto-ot, Peixa pedra, Pek chor, Perche argentée, Pullikurimeen, Rokok, Roncador-prateado, Rupali datina, Samu-ok, Sasavy, Sebokoh, Sebokok, Selukut, Serkut, Silver grunt, Silver grunter, Silver javelin, Silver spotted grunt, Silverlined grunt, Silverlined grunter, Small-spotted grunter-bream, Small-spotted javelin fish, Sohisohy, Spotted javelinfish, Srebrna prašičevka, Sølvgryntefisk, Tabal, Taguk-guk, Tau lo, Thondakappe, Thondo, Trumpeter, Täpiline uriseja, Ulibalay, White-finned javelin fish | 413 |
| *Pomadasys kaakan* | Javelin grunter | Aguut, Ambah, Barred javelin, Celak mata, Common javelinfish, Gerut-gerut, Gerut-gerut ompakan, Grondeur javelot, Grunter, Hta-min-byone, Huuqle, Ibalay, Ikan kepala batu, Ikuran, Javelin grunt, Javelin grunter, Ka-la-khote, Karamamba, Kepala batu, Kepala besar, Khruetkhrat, Kiskisan, Likti, Lined silver grunter, Nagroor, Nagrur, Nga-khone, Ompak, Peh choe, Peixe pedra, Queensland trumpeter, Rokok, San-hla-may, Sangsar-e-maamooli, Sebokoh, Selukut, Serkut, Si kerekere, Siget, Silver grunt, Spies-knorder, Spotted grunter-bream, Spotted javelinfish, Spotted-grunter bream, Talun kinur, Talun mutin, Trumpeter, Ulibalay, Umpak, Yellow-finned javelin fish | 6319 |
| *Psammogobius biocellatus* | Neuli Baligirida | Bia, Bia, Busongchut, Cá Bống mấu mắt, Estuary goby, Gobi de tas, Gobie biocellé, Gobie à deux taches, Hitomihaze, Kora, Krokotiilitokko, Mangrove goby, Mano'o, Mollon, Mulug, Phursandi, Ploso, Sleepy goby, Two spot goby, Vaak dikkop | 10269 |
| *Rhabdosargus sarba* | Dhala Khuranti | Ambatovasena, Brumbrufuma, Brème de mer, Chitchillee, Cá Tráp dẹp, Dourada, Dourada comum, Fihampotsy, Fihampotsy, Gabit, Gold-line sea bream, Gorgofan, Gueule pavée, Guldlinjet havrude, Haffar, Hedai, Jerjafan, Kabeet, Kapas batu, Kifo, Menaheliky, Mengkuku garis emas, Natal stumpnose, Natalse stompneus, Palu, Saifotsy, Sampea, Sargo dorado, Sargue doré, Sargus sarba, Seabream, Silver bream, Silver sea bream, Tarwhine, Tsaralela, Vahoho, Vella-mattawa, Xinguende, Yellow fin bream | 5624 |
| *Scatophagus argus* | Chitra chandi | Akikiro, Almindelig Argusfisk, Argus, Argus fish, Argusfisch, Argusfisk, Arguskala, Baba, Baladhan, Bannsire, Baraayi, Bat hu, Bayang, Bidang, Bishtara, Bushami, Butter fish, Butterfish, Buurrun, Buurrunbul, Chandar, Chitsillo, Chubei, Common scat, Cá nau, Cá nào, Cá Nâu, Cá nú, Cá Nầu, Eesputti, Gam ku, Gemeiner Argusfisch, Grüner Argusfisch, Harilik roojakala, Ilattiya, Kapiged, Kaski, Kendang, Keper, Kero kero, Kertang, Ketang-ketang, Kikero, Kikilo, Kikiro, Kitang, Kitong, Kurohoshimanjûdai, Lanbia, Lankia, Leopard scat, Malaga, Mia mia, Nachikarimeen, Nga-khet, Nga-pathown, Ngi-ni-ngisi, Ngisi-ngisi, Nutchar-char, Pavillon tacheté, Peebe, Pingaw, Pingo, Pingo manchado, Plettet argusfisk, Quequero, Samaral, Scat, Sipili, Spade fish, Spadefish, Spotted butter fish, Spotted scad, Sungeli, Takrap, Tiger butterfish, Tiger seat, Tikero, Titang, Vada, Vadda, Veita kau, Vetakau, Zarook | 4919 |
| *Rhinomugil corsula* | Corsula mullet | Corsula, Corsula mullet, Corsula-multe, Indische Meeräsche, Karsul, Khorsula, Korsula, Mizhugu meen, Mullet, Nga-kin, Ural | 5577 |
| *Siganus javus* | Streaked spinefoot | Alama, Balawis, Barangan, Batataway, Bataway, Batid, Batwayi, Belais, Belait, Belaris, Belibas, Beronang, Bliais, Blue-spotted spinefoot, Blue-spotted trevally, Bluespotted spinefoot, Bonang-bonang, Borras-Danggit, Bulawis, Buras, Cà Dìa xanh, Danggit, Danggit, Dangit, Dayagbagu, Debam, Debam leban, Dengkis, Dengkis Jawa, Dengkis leban, Gelibas, Ginava, Indongan, Java rabbitfish, Java spinefoot, Java-kaninfisk, Kelang, Ketang, Ketang lada, Kitang, Kitong, Kunar, Lada, Lambai, Layap,Leban, Limaran, Lumban, Lusay, Malaga, Manaring, Mandalada, Moblad, Mublad, Nava, Nga-suee-than, Nga-yan-shar, Ottah, Padas, Rabbitfish, Safi, Safi senefi, Safi-e-modjar, Salitthalethaep, Samaral, Seeseege, Sigan ondulé, Sigano ondulado, Spinefoot, Streaked spinefoot, Suputik, Tabago, Tagbago, Tambago, Taragbago, Tayog, Toros, Turos, Turus, Vori, Warahwah, White-spotted rabbit-fish | 4805 |
| *Sillago sihama* | Silver sillago | Ago-os, Alisoos, Amborody, Amboso, Ambotroka, Ambotso, Ambotsoka, Arriti-ki, Aso-os, Asohos, Asoos, Asos, Asu-os, Asuhos, Asuos, Asus, Awsaos, Bangalus, Bebolos, Bebulus, Beritus, Besot, Bojor, Bulus, Bulus-bulus, Bunus, Caanood, Chonsaikaeo, Common asohos, Common sillaginid, Common whiting, Cudeerah, Culingah, Cá Đục bạc, Harilik liivaahven, Hasoom, Hasum, Hosohos, Ili, Indian whiting, Kalanda, Kane, Kedondong-kedondong, Kelakkan, Kelangan, Kilaken, Kilangan, Kisu, Koryushkovaya sillaga, Kunga, Moto-gisu, Mtambaanchi, Mudadi, Muxile, Nga-pa-lwae, Nga-pa-lway, Nga-palwe, Nga-thae-htoe, Nga-zan-pu, Ngulu, Noongal, Northern whiting, Oso-os, Osoos, Osu-us, Pescadinha branca, Pescadinha comum, Poovan, Pooyan, Poozhan, Punting damar, Puntung-damar perak, Pêche madame, Pêche-madame argenté, Rejun(g), Renvi, Sale-mwinyi, Sand border, Sand smelt, Sand whiting, Shoort, Shorangi, Sigarilyohon, Silago plateado, Silagos, Sillago-whiting, Sillago-whitings, Silver sillago, Silver whiting, Silwer sillago, Smelt, Sondo, Soos, Soring, Suar chiam, Suar chooi, Susay, Swam, Sylagus smukly, Tambanji, Tayutos, Tella-soring, Toholava, Toul-danti, Trumpeter whiting, Ubi, Usaos, Usu-us, Usus, Warijung, Whiting | 4729 |
| *Stolephorus commersonnii* | Commerson’s anchovy | Ake lae, Anchois bombra, Anchoveta de Commerson, Anchovy, Bali-bali, Balingon, Barriya, Besoin, Bilis, Bilis tembaga, Bolinao, Bombra, Boquerón bombra, Bulinao, Bulinaw, Bunga air, Burma, Chwali-patwa, Commerson's anchovy, Cá Cơm thướng, Dagaa-mcheli, Devis's anchovy, Dilis, Gurayan, Hagongan, Hal massa, Halmassa, Kang hu, Kella, Kolla, Long-jawed anchovy, Mempinis teri, Muleng-leng, Munamom, Neplie, Nethali-netholi, Nethili, Netholi, Nga-nan-gyaung, Purasa, Pusu, Saitan, Samaduul, Sardel commersonova, Sardelina komersonka, Teri, Teri anchovy, Tigih, Tornos, Tropical anchovy, Tuakang, Tumbagan, Tuwakan, Tuwakang, Uono, Zaam | 582 |
| *Stolephorus indicus* | Indian anchovy | Alipatang, Anchois indien, Anchoveta do Indico, Anchovy, Anszowetka indyjska, Balinau, Balingon, Barriya, Bavangawan, Besin, Bilis, Bilis bunga air, Bilis paku, Bolinao, Bolinao na puti, Bolinaw, Bombra, Boquerón indio, Boris, Bulinao, Bulinaw, Bunga air, Burma, Ca-soc-phan, Conetholi, Cá Cơm Ấn Độ, Dagaa-mcheli, Dilis, Dilis tantakot, Dindus, Dumudot, Guno, Gurayan, Halmassa, Handalla, Hardenberg's anchovy, India stolefoor, Indian anchovy, Indiese ansjovis, Indo-ainoko-iwashi, Kang hu, Katakyai, Lambiyang, Libgaw, Lipatang, Matalos, Mempinis teri, Motoo hendi, Muleng-leng, Muleng-leng, Nattoo, Nehu, Nethili, Nga-ni-tu, Puri putih, Pusu, Samaduul, Sardel indická, Silag, Teri, Tigih, Tinggih, Tornos, Trwi-bao, Tuakang, Tuarang, Tuwakang, Yae-kyin-ngae, Zaam | 585 |
| *Strongylura strongylura* | Gania | Agujón ocelado, Aiguillette ocellée, Ayakorakoli, Balo, Balu, Balô, Banded needlefish, Batalay, Batali, Bigiw, Bilan, Blackspot longtom, Cencodak, Chernokhvostyi sargan, Coomeen, Coplah, Cá Nhói đuôi chấm, Dethuntholi, Dhiya moralla, Dual, Full beak, Full beak Gar fish, Gania, Gar fish, Garfish, Hagul, Hakul, Hamalit, Jolong-jolong, Kagada, Kanda, Karkor, Kathungkhwai, Keran, Konthe, Kuddera, Kunga, Light-colored garfish, Loncong, Marakola, Menyulong, Moakoli, Mustsaba-tuulekala, Neddlefish, Nedumurrel, Oola, Ooshee-colarchee, Ooshee-collarchee, Pallankoil, Pambankoli, Pambu mural, Plethalet hornfisk, Round-tail alligator garfish, Roundtail needlefish, Salasa, Sathamurrel, Sigliwan, Siriw, Spottail needlefish, Subingan, Sumb, Surali, Tambilawang, Todak, Todak belang, Tol, Vadyamurrel, Vellaimurrel, Wadlah muku, Wahleh kuddera, Wodlah-muku | 1334 |
| *Systomus sarana* | Olive barb | Bada pothi, Bhitti, Darai, Didpakke, Doodha paraga, Gende, Kande, Kannaku, Kurichi, Kuruka, Mas pethiya, Munduttee, Oliivjas saagpuntius, Olive barb, Paraga, Parma bhútánská, Parmička pondičerská, Peninsular olive barb, Pondicherry barb, Pullan, Pungella, Sar puti, Sarpunti, Shorpunti, Tapien | 13523 |
| *Terapon jarbua* | Gahana | Antsarabaro, Ava'ava, Baam, Babaguni, Bagaong, Bagaong kambang, Bagat, Bagaw, Baikeeli, Balauling, Banlaongan, Baraonga jarbúa, Baraongan, Baraungan, Bigaong, Bobi, Bogaong, Bongaong, Boorgooni, Borguni, Buga-ong, Bugaong, Chavathinnikeeli, Convex-lined grunt, Convex-lined therapon, Crescent grunter, Crescent-banded grunter, Crescent-banded tiger-fish, Cresent grunter, Cresent perch, Croaker, Cá Căng cát, Cá ham, Cá Ong, Dheeb, Dhiba, Dirhar, Dogigi, Doringvis, Drihe sak, Drihy, Gagaong, Gahnu, Galikooru, Ganam, Garangeta, Gawaany, Geedow, Gelama, Gendang-gendang, Gendarme, Gonga, Gore, Grunter, Grunzender Tigerbarsch, Gunggong, Habraham, Henw, Hyoaak, Iri bateya, Jambrung, Jangjan, Janjan, Jarbua, Jarbua terapon, Jarbua therapon, Jarbua tigerfish, Jeerpye, Jimjam, Kaboa, Kalaero, Kallakeedam, Kanigit, Karleku, Keechan, Keeli, Keeri, Kelong-kelong, Kerong jalur, Kerong-kerong, Kerong-kerong tambi, Kerung-kerung, Keskus, Khangtaphaolaikhong, Kili, Kilipothu, Kirong-kirong, Kliklechol, Kokoreh, Kolmvööt-tiigerahven, Kotha, Kotohiki, Kourmanoue, Kourmaoue, Kovakeechan, Kui, Kurmanwe, Lakeke, Langaat, Mangahua, Marakkeri, Mattakkeechan, Mayavayawong, Mengkerong, Mwârâgiya, Mwââgié, Naida, Naveri, Naveri hajam, Nga-gone-kyar, Nga-hnan-kyang, Nga-khone-kyar, Nga-nan-gyaung, Ngagu, Palin-keetchan, Palinkeechan, Peau d'âne, Peixe-zebra violão, Pol bateya, Pootankeeli, Qitawa, Relégué, Rongkador, Rumpak, Sal-ben-ja-ri, Samudrakili, San gaa, Sekirong, Spiky trumpeter, Squeaking perch, Sukiroeng, Sumaha, Theeb, Thonfish, Thornfish, Three-striped tiger fish, Threestripe tigerfish, Tiger perch, Tsarabaro, Tutot, Violon, Violon jarbua, Xì lín là, Yagata-isaki, Yalli, Yalli-e-khatkamani, Yamyam, Yanam, Yoxany | 4656 |
| *Tenualosa ilisha* | Ilishi | Alose hilsa, Brk, Chakshi, Chaksi, Cá Cháy, Dolum, Hilsa, Hilsa herring, Hilsa shad, Hilsa-stamsild, Hilza indyjska, Ilish, Ilisha, Ilishmach, India salealoosa, Indijska vitka čepa, Indisk staksill, Jatka, Jodi, Karuva-ullam, Mahi Khor Kuchiku, Mullasu, Nga-tha-lauk, Nga-thalank, Oolum, Pala, Palasa, Palasah, Palia, Paliya, Paliyah, Palla, Palo, Paluva, Palva, Pepe, Placka iliša, Polasa, Pulla, Sabur, Sevva, Shour, Sleď palasah, Soboor, Sobur, Suboor, Sábalo hilsa, Ullam, Valava, Zabur, Zomur, Si kerekere, Small-scaled terapon, | 1789 |
| *Tricanthus biaculeatus* | Sukura | Anjungkang, Ankatilla, Barat-barat, Black-finned triple-spine, Blacktail tripodfish, Butiti, Cagak langit, Chamat, Cá bò, Cá Bò ba gai đầu nhọn, Gima, Harilik kolmoga, Hollow-snouted tripodfish, Ikan lembu, Kissii, Kortsnudet tretornfisk, Lembu muncung, Mullu klathai, Panisan, Papakul, Petek, Saguksok, Se-khareh-e-poozeh-kootah, Sereka, Shalaib ad-dau, Short nosed tripod fish, Short-nosed tripodfish, Shortnose tripodfish, Shuāng jí sān cì tún, Sokang, Songo langit, Sula bagyo, Sulay-bagyo, Sungay-sungayan, Sunyang langit, Thun katuva, Tikos, Tripod fish, Tripodin nez court, Tripodín ñato, Trójkolec niebieski, Tukod langit, Tunjak langit, Wuachamuksan | 4747 |
| *Wallago attu* | Balia | Athiyala, Attu vaalay, Attu-valai, Attuvaala, Baalae, Bahle, Balae, Ballai, Balu shivada, Barali, Barwari, Bayali, Boyari, Cangop, Chate, Cá leo, Freshwater shark, Giant sheatfish, Godlay, Hubschrauber-Wels, Intianpetomonni, , Jambal, Khao, Koyali, Kropaut, Mully, Nga-bat, Nga-bat, Padin, Padni, Pahree, Paran, Parhin, Pari, Pattan, Pla khao, Poil, Pozha wallah, Purram, Sandai, Sareng, Shark catfish, Shivada, Tapah, Trey kråpoat, Trey sanday, Valaga, Valai, Valashivda, Valuga, Vazhai, Walagah, Walaya, Wallagah, Wallago, Wallah, Warshoorah, Whiskered Catfish | 10567 |
